# Extracellular ATP Mediates Cancer Cell Migration and Invasion Through Increased Expression of Cyclooxygenase 2

**DOI:** 10.3389/fphar.2020.617211

**Published:** 2021-01-27

**Authors:** Shilpa Sharma, Harshit Kalra, Ravi Shankar Akundi

**Affiliations:** Neuroinflammation Research Lab, Faculty of Life Sciences and Biotechnology, South Asian University, New Delhi, India

**Keywords:** cancer recurrence, COX-2, metastasis, P2 receptor, PBAIDs, prostaglandin E2, tumor microenvironment, residual ATP

## Abstract

The tumor microenvironment plays a major role in the ability of the tumor cells to undergo metastasis. A major player of tumors gaining metastatic property is the inflammatory protein, cyclooxygenase 2 (COX-2). Several tumors show upregulation of this protein, which has been implicated in mediating metastasis in various cancer types such as of colon, breast and lung. In this report, we show that the concentration of extracellular ATP (eATP) is increased in response to cell death mediated by chemotherapeutic agents such as doxorubicin. By using three different cell-lines—HeLa (cervical), IMR-32 (neuronal) and MCF-7 (breast)—we show that this eATP goes on to act on purinergic (P2) receptors. Among the various P2 receptors expressed in these cells we identified P2X7, in IMR-32 and MCF-7 cells, and P2Y12, in HeLa cells, as important in modulating cell migration and invasion. Downstream of the P2 receptor activation, both p42/44 mitogen-activated protein kinase (MAPK) and the p38 MAPK are activated in these cells. These result in an increase in the expression of COX-2 mRNA and protein. We also observe an increase in the activity of matrix metalloproteinase 2 (MMP-2) enzyme in these cells. Blocking the P2 receptors not only blocks migration and invasion, but also COX-2 synthesis and MMP-2 activity. Our results show the link between purinergic receptors and COX-2 expression. Increased levels of ATP in the tumor microenvironment, therefore, leads to increased COX-2 expression, which, in turn, affords migratory and invasive properties to the tumor. This provides P2 receptor-based anti-inflammatory drugs (PBAIDs) a potential opportunity to be explored as cancer therapeutics.

## Introduction

Globally, the incidences of cancer detection are on the rise with more than 9.6 million deaths in 2018 alone (Bray et al., 2018). Despite advances in medical research, and an increase in the number of treatable cancers, it still remains one of the deadliest diseases worldwide with recurrence of cancer in treated patients being a major concern (Houssami et al., 2014; Holleczek et al., 2019). While the actual causes of tumor recurrence is not known, it is dependent on tumor size and its microenvironment (Avanzini and Antal, 2019). The tumor microenvironment has a range of inflammatory molecules that affect its progression and metastasis depending on the stage of the tumor and the immune phenotype (Wang and DuBois, 2015). One of these inflammatory modulators is the enzyme cyclooxygenase 2 (COX-2), responsible for the synthesis and release of prostaglandin E_2_ (PGE_2_) (Wang et al., 2007; Nakanishi and Rosenberg, 2013). The role of COX-2 in inflammation is well established (Akundi et al., 2005; Minghetti, 2007). In recent years, its role in cancer has also been observed, especially related to tumors acquiring metastatic potential and chemotherapy resistance (Ferrandina et al., 2002; Li et al., 2002). Many cancer types such as leukemia, breast cancer, pancreatic cancer, lung cancer and lymphomas show overexpression of COX-2 (Nakanishi et al., 2001; Ristimaki et al., 2002; Wun et al., 2004; Secchiero et al., 2005). This increase in COX-2 has been shown to enhance tumor progression through increased apoptosis resistance and increased metastatic properties (Choi et al., 2005; Singh et al., 2007; Karavitis and Zhang, 2013). Gonadotropins such as follicle-stimulating hormone or luteinizing hormone have been shown to increase migration and invasion in ovarian cancer cells via COX-2 (Feng et al., 2017). Although the nuclear factor κB (NF-κB) pathway has been implicated in the increased expression of COX-2 in cancer cells (Kim et al., 2014; Kuang et al., 2017), further upstream causes are not completely known.

On the other hand, several reports support the role of purinergic receptors in modulating tumor growth (Deli and Csernoch, 2008; Di Virgilio et al., 2018). Therapeutic interventions such as irradiation and chemotherapy have been shown to increase the levels of extracellular nucleotides in several organs (Schneider et al., 2015). Under normal physiological conditions, although there is only a few nmol/L concentration of ATP in the extracellular space, it has been shown to increase to several mmol/L levels under physiological stress such as hypoxia, inflammation and cancer (Khakh and North, 2006; Idzko et al., 2014). In addition to a non-regulated release of ATP from dying/damaged cells, active release of ATP also occurs through exocytic granules, microvesicles and various transporters and channels located on the tumor cells (Vultaggio-Poma et al., 2020). The role such huge amounts of extracellular ATP (eATP) may play in the tumor microenvironment has been debated with both pro- and anti-tumourogenic outcomes (Jiang et al., 2015; Avanzato et al., 2016; Pavlovic et al., 2020). This outcome largely depends on the type of receptor eATP acts on, of which there are ion-channel associated P2X receptors, G protein-coupled P2Y receptors, and adenosine-specific adenosine receptors (Fiebich et al., 2014). Expression of these purinergic receptors is found to be higher in tumor cells, thereby making them relevant to tumor progression (Di Virgilio et al., 2018). The type of P2 receptor expressed and the concentration of ATP in the microenvironment, which in turn is dependent on the activity of ectonucleotidases (Mandapathil et al., 2018; De Marchi et al., 2019; Soleimani et al., 2019), determine the progression of tumor. How the purinergic receptor activation mediates tumor progression is not completely known. ATP released by chemotherapy-sensitive cells can have an immunosuppressive function; thereby assisting tumor cells evade immune surveillance (Lecciso et al., 2017). However, mechanisms through which eATP enhances tumor progression remains to be addressed.

We have previously hypothesized that eATP released by dying cells acts as a second hit during inflammation leading to enhanced expression of COX-2 (Fiebich et al., 2014). In this study, we have extended this hypothesis to tumor cell progression. We propose that the high concentration of eATP in the tumor microenvironment is responsible for the increased COX-2 expression in tumor cells leading to their attaining metastatic properties. We show this through the use of different P2 receptor agonists and antagonists on tumor cell migration and invasion and expression of COX-2 in these cells. Our report, for the first time, provides evidence of the link between increased purinergic receptor expression in tumor cells and of increased COX-2 expression which is responsible for imparting metastasis.

## Materials and Methods

### Cell Culture and Reagents

All reagents were purchased from Sigma Aldrich (Bengaluru, India), HiChem Life Sciences (Ghaziabad, India), or Fisher Thermo Scientific India (Mumbai, India). The human cervical cancer cell line, HeLa (kindly provided by Dr Yubaraj Pokharel, South Asian University, New Delhi, India), the breast cancer cell line, MCF-7 (kindly provided by Dr Seema Sehrawat, Shiv Nadar University, Dadri, India) and the human neuroblastoma cell-line, IMR-32 (from National Center for Cell Science, Pune, India), were cultured in Dulbecco’s MEM supplemented with 100 U/ml penicillin, 100 μg/ml streptomycin, 25 μg/ml amphotericin B, 1 mM sodium pyruvate, 2 mM l-glutamine and 10% heat-inactivated fetal calf serum (HiChem Life Sciences). Cells were cultured at a density of 5 × 10^4^ cells/cm^2^ in various formats (6-, 24- or 96-well plates) as per experimental requirement and were stimulated the following day when the cells reached ∼70% confluency. Media was changed an hour prior to stimulation with the inhibitors added 30 min prior to the addition of ATP or P2 receptor agonists. ATP, apyrase and suramin were from Sigma Aldrich while all other P2 receptor agonists and antagonists, MAPK inhibitors, and COX inhibitors were obtained from Tocris Biosciences (Bio-Techne India, Pune, India). Doxorubicin was bought from HiMedia (HiChem Life Sciences).

### Cell Death Analysis

HeLa, IMR-32, and MCF-7 cells were stimulated with 200 nM doxorubicin for 48 h. Cells were then washed, resuspended in ice-cold phosphate-buffered saline (PBS) and fixed in ethanol. Cells were then stained with propidium iodide (PI, 20 μg/ml) in PBS for 10 min. PI-positive cells, indicating apoptosis, were counted on a flow cytometer and analyzed using BD FACS Suite software (BD Biosciences, San Jose, CA).

### Quantitation of ATP Release

Extracellular ATP was measured in the culture supernatants of different cells treated with doxorubicin for 48 h. A bioluminescent ATP assay kit (Promega, New Delhi, India) was employed as per the manufacturer’s instructions. Luminescence was recorded with a microplate reader (Synergy HT Biotek). To calculate the concentration of ATP released in the supernatant, an ATP standard curve of 0.78–500 nM range was employed.

### Wound Healing Assay

Wound healing or scratch assay was performed as described elsewhere (Liang et al., 2007). Cells were plated in a 6-well plate and allowed to form a uniform monolayer. Scratch was made using a 200 µl tip in each well. The scratched wells were washed twice using media prior to treatment. The zero hour pictures of 6 random scratch areas were taken using a Nikon microscope. The plates were incubated in a humidified 5% CO_2_ incubator kept at 37 °C and the same areas were imaged at different time intervals. For analysis percentage area healed was calculated using NIH ImageJ software (https://imagej.nih.gov/ij).

### *In vitro* Migration Assay

Migration assay was performed as described elsewhere in a 24-well plate wherein Transwell inserts (Corning) of 8 μm pore size were placed (Liang et al., 2007). Cells were seeded at a density of 10,000 cells/insert on the upper chamber in serum-free media. Complete media containing the respective treatment was poured in the lower chamber of the transwell setup. At the end of the incubation point (27 h for HeLa, 18 h for IMR-32, or 12 h for MCF-7 cells), cells on the upper chamber were scrapped while the migrated cells from the lower side of the membrane were fixed in 70% ethanol and stained with 1 mg/ml Hoechst 44,432 for 5 min. The stained cells were imaged under a fluorescence microscope and counted using the NIH ImageJ software (https://imagej.nih.gov/ij).

### Cell Invasion Assay

Transwell migration assay was modified using 0.1 mg/ml matrigel matrix (Corning) coating. 20,000 cells were plated above the matrigel coating in the transwell insert and allowed to invade along the treatment gradient. At the end of incubation (27 h for HeLa, 18 h for IMR-32, or 12 h for MCF-7 cells), cells on the upper chamber were scrapped while the invaded cells from the lower side of the membrane were fixed in 70% ethanol and stained with 1 mg/ml Hoechst 44,432 for 5 min. The stained cells were imaged and counted as described above.

### Western Blot

Total cell lysates were prepared in a lysis buffer composed of 42 mM Tris-HCl, pH 6.8, 1.3% (w/v) sodium dodecylsulfate, 6.5% glycerol, 0.1 mM sodium orthovanadate, and protease inhibitor cocktail (from Sigma-Aldrich). Protein content was measured using the bicinchoninic acid method (Thermo Fisher Scientific) using bovine serum albumin (BSA) as standard. 2-Mercaptoethanol (final concentration 1%) and bromophenol blue (0.2 mg/ml) were added to the samples and heated at 95 °C for 5 min before electrophoresis. In total, 20–50 μg samples were loaded on a 7.5% (for COX-2 and MMP-2) or 12% (for p38 and p42/44 MAPK) polyacrylamide gel under reducing conditions. Separated proteins were transferred onto a polyvinylidene fluoride membrane (Merck LifeSciences, Mumbai, India) and blocked for 1 h with 5% BSA in Tris-buffered saline containing 0.1% Tween-20 (TBS-T) followed by primary antibody at 4 °C overnight. Primary antibodies used were rabbit anti-COX-2, rabbit anti-MMP-2, rabbit anti-phospho-p42/44 MAPK (detecting endogenous levels of p42/44 only when dually phosphorylated at Thr^202^ and Tyr^204^ of Erk1 and Thr^185^ and Tyr^187^ of Erk2), rabbit anti-phospho-p38 MAPK (detecting endogenous levels of p38 MAPK only when phosphorylated at Thr^180^ and/or Tyr^182^), rabbit anti-p42/44 MAPK, and rabbit anti-p38 MAPK (all from Cell Signaling Tech, Danvers, MA, United States and used at 1:1,000 dilution in TBS-T containing 1% BSA). For the normalization of protein loaded, mouse anti-β-actin (Sigma-Aldrich) was used at 1:5,000 dilution. Secondary antibody was diluted 1:10,000 in 1% BSA in TBS-T for 1 h at RT and washed extensively. Proteins were detected using chemiluminescent solution made by mixing equal volumes of solution A (2.5 mM luminol, 0.396 mM p-coumaric acid and 0.1 M Tris-HCl, pH 8.5) and solution B (5.2 mM H_2_O_2_ and 0.1 M Tris-HCl pH 8.5).

### Gelatin Zymography

The proteolytic activity of matrix metalloproteinase (MMP-2) was analyzed by substrate-gel electrophoresis method as described elsewhere (Toth et al., 2012). Briefly, cells were plated in 6-well plates in complete media while treatment was given under serum-free conditions. The culture supernatant was collected, mixed with non-reducing dye and run on a SDS-PAGE gel containing 0.4% (m/v) gelatin. At the end of electrophoresis, the gels were washed in a buffer comprising of 2.5% Triton X-100, 5 mM calcium chloride, and 1 μM zinc chloride and incubated for 16–27 h in the activation buffer (1% Triton X-100, 0.2 M sodium chloride, 5 mM calcium chloride, and 1 μM zinc chloride). The gels were then stained with Coomassie blue wherein the presence of any clear white bands were indicative of the gelatinolytic activity of MMP-2.

### RNA Isolation and Real Time PCR

Total cellular RNA was isolated from cells using Trizol reagent (Fisher ThermoScientific) as per the manufacturer’s instructions. 2 μg total RNA was used for cDNA synthesis using the PrimeScript first strand cDNA Synthesis kit (DSS Takara, Advaita Biosciences). 1 μl of cDNA sample was used for real time PCR using SYBR Green Master Mix and 0.2 μM each of forward and reverse primers which were designed through Primer Blast (https://www.ncbi.nlm.nih.gov/tools/primer-blast/) and ordered through a commercial vendor (IDT). Sequences for the primers are tabulated in Table 1. The reactions were carried out in Applied Biosystem ViiA™ 7 system and mRNA expression analyzed using the δδC_t_ method. Fold change was calculated as 2^−δδC^_t_ where δC_t_ is the difference between the C_t_ values of COX-2/P2 receptor and β-actin for each cDNA sample and δδC_t_ is the difference between the δC_t_ values of stimulated condition (ATP and its agonists along with P2 blockers) with that of the unstimulated one (control).

**TABLE 1:** List of primers used in the study.

Gene		Primer Sequence	Gene ID
P2X1	F	5′-TAC​GTG​GTG​CAA​GAG​TCA​GG-3′	1519314411
	R	5′-CCA​GGT​CAC​AGT​GCC​AGT​C-3′	
P2X4	F	5′-GCT​TTC​AAC​GGG​TCT​GTC​A-3′	330448876
	R	5′-AGT​GAA​GTT​TTC​TGC​AGC​CTT​T-3′	
P2X7	F	5′-GCG​GTT​GTG​TCC​CGA​GTA​T-3′	1854511
	R	5′-CCT​TCC​GGT​CTG​AAT​TCC​TT-3′	
P2Y1	F	5′-CCC​GAA​ACT​GAG​CTG​CAC-3′	1519314089
	R	5′-TCA​ACT​TAA​TTG​GGG​CAT​C-3′	
P2Y2	F	5′-CCT​CAA​GAC​CTG​GAA​TGC​GT-3′	20380402
	R	5′-GTA​ATA​GAC​CAG​CAG​CGG​CA-3′	
P2Y4	F	5′-TGG​CAG​TTT​GGT​TGG​TCG​TA-3′	109730011
	R	5′-TGG​TCC​CTT​TGT​TGC​TGG​TT-3′	
P2Y6	F	5′-CAC​CCA​CCA​CCT​GTG​TCT​AC-3′	1407632
	R	5′-ACA​CAG​ATG​TTC​AGC​GGC​AG-3′	
P2Y12	F	5′-CTC​TCT​GTT​GTC​ATC​TGG​GCA-3′	17389766
	R	5′-GCT​GCC​TGT​TGG​TCA​GAA​TC-3′	
COX-2	F	5′-TGA​GCA​TCT​ACG​GTT​TGC​TG-3′	80,142
	R	5′-ATC​ATC​AGA​CCA​GGC​ACC​A-3′	
β-Actin	F	5′-CCA​ACC​GCG​AGA​AGA​TGA-3′	178023
	R	5′-CCA​GAG​GCG​TAC​AGG​GA-3′	

### Statistical Analysis

All experiments were done in triplicates with each experiment made on cultures derived from at least three different thawed vials for each cell-line. Migration and invasion analyses were counted from at least 15 different fields per transwell from each passage. Normal distribution of data was assessed using the Shapiro-Wilk test. Data are presented as mean ± standard error of the mean (SEM) and were analyzed using Student’s *t*-test, using Microsoft Excel software. Differences were considered significant when *p* < 0.05.

## Results

### Doxorubicin Increases Levels of Extracellular ATP in Correlation to Cell Death

**FIGURE 1:**
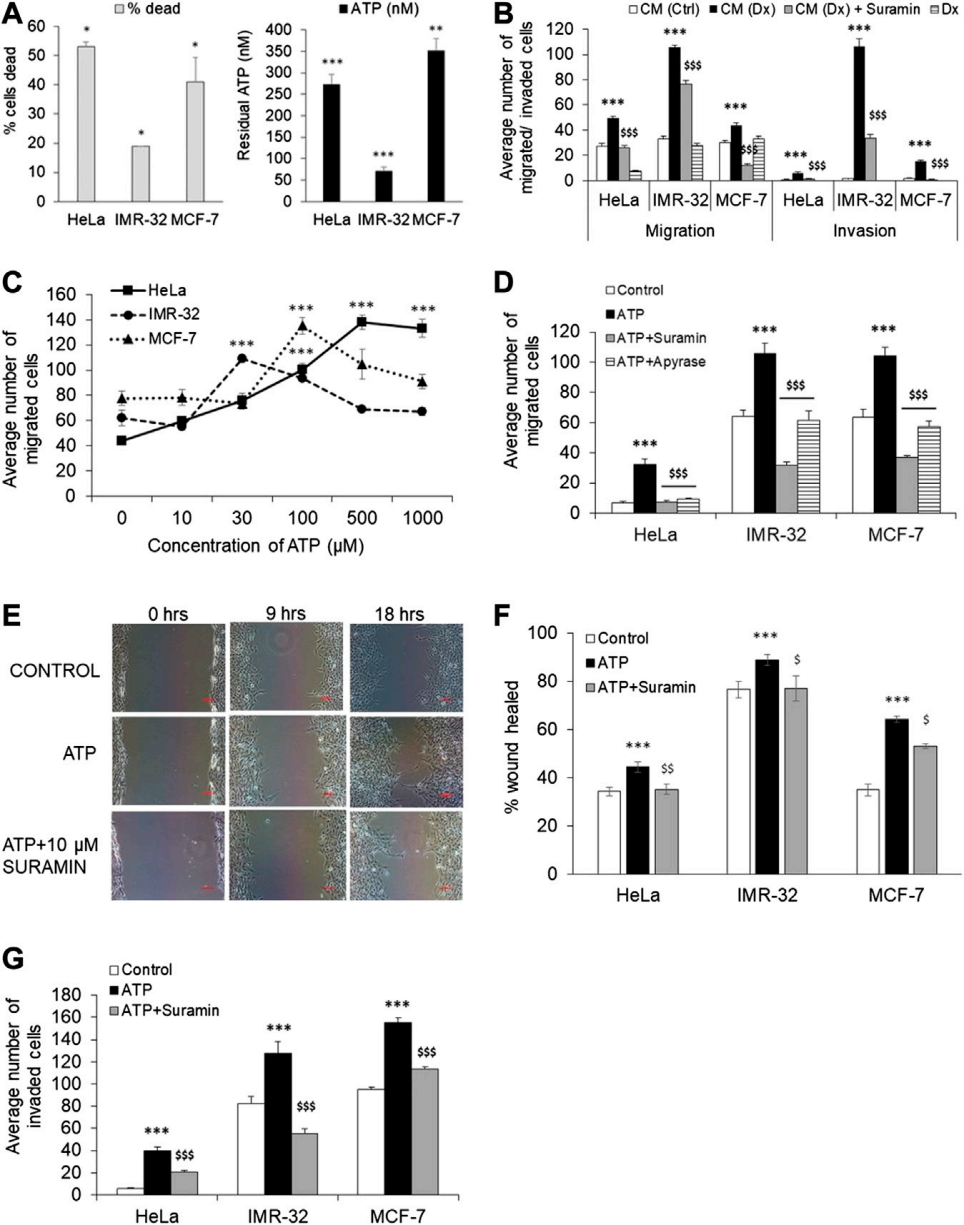
Extracellular ATP enhances cancer cell migration and invasion. **(A)** HeLa, IMR-32 and MCF-7 cells were treated with 200 nM doxorubicin for 48 h following which the media was collected for measuring ATP levels (black bars) while the cells were stained with propidium iodide for cell death analysis through flow cytometry (gray bars). 10,000 cells (for HeLa and MCF-7) and 30,000 cells (for IMR-32) were run on a flow cytometer and the % cells dead depicted in gray bars. The amount of ATP released in the media was calculated based on a standard curve and represented here (*n* = 3 independent experiments). Data depicted mean ± SEM. *p < 0.05, **p < 0.01, ***p < 0.001 by Student’s *t*-test. **(B)** Cell migration was assessed by culturing cells on top of a transwell insert in serum-free media while media containing serum was placed in the lower chamber. The same set up was used for invasion studies wherein the cells were instead plated on top of a matrigel in the transwell insert. Migration/invasion was analyzed 24 h after the addition in the lower chamber media from untreated HeLa cells (CM-Ctrl), from HeLa cells treated with 200 nM doxorubicin (CM-Dx), CM-Dx with addition of 10 μM suramin, or only doxorubicin (Dx). Cell counts from the lower chamber are depicted as mean ± SEM and are from three independent experiments. ***p < 0.001 with respect to cells treated with CM-Ctrl, ^$$$^ *p* < 0.001 with respect to cells treated with CM-Dx, for the same cell type, by Student’s *t*-test. **(C)** Migration assay as described in **(B)** was performed in each cell-line stimulated with different concentration of ATP. The number of migrated cells to the bottom of the Transwell were counted and depicted as mean ± SEM. ***p < 0.001 with respect to unstimulated cells of the same type. **(D)** The migration assay as described in **(B)** was performed in each cell-line which were stimulated with ATP (1 mM for HeLa, 0.1 mM for MCF-7, and 30 μM for IMR-32) in the presence or absence of 10 μM suramin or 5 U/ml apyrase. Cell counts were done as in **(B)**. ***p < 0.001 with respect to unstimulated cells, ^$$$^ *p* < 0.001 with respect to ATP-treated cells of the same type. **(E)** Representative images of the wound healing assay in IMR-32 cells is depicted. Scale bar = 100 μ. **(F)** Confluent cultures of HeLa, IMR-32 or MCF-7 were subjected to a scratch followed by stimulation with ATP as mentioned in **(D)** above, alone or together with 10 μM suramin. The percentage of the wound healed after 27 h (for HeLa), 18 h (IMR-32) or 12 h (MCF-7) is depicted here. Paired Student’s *t*-test shows significance of *p* < 0.001 with respect to control (***) or ATP-treated (^$^ *p* < 0.05, ^$$^ *p* < 0.01) cells for the respective cell-type (*n* = 5 independent experiments). **(G)** Cell invasion assay was conducted as mentioned in **(B)** with cells treated with ATP, alone or together with 10 μM suramin, as in **(F)**. Paired *t*-test shows significance of *p* < 0.001 with respect to control (***) or ATP-treated (^$$$^) cells.

We had hypothesized that during chemotherapy drug-sensitive cells undergo cell death wherein their cellular contents, including ATP, are released into the microenvironment. To test this hypothesis, we checked the effect of the common chemotherapeutic agent, doxorubicin, on three different cell-lines—HeLa (cervical), IMR-32 (neuronal) and MCF-7 (breast). Flow cytometric data showed that at 200 nM dose, doxorubicin mediated 19% cell death in IMR-32 cells, 41% death in MCF-7 cells and 53% death in HeLa cells, 48 h post-stimulation (Figure 1A). Corresponding to this increase in cell death, there was also a concomitant increase in the levels of extracellular ATP (eATP) in the culture media in all 3 cases. In the case of HeLa cells, this corresponded to an increase to 272 ± 25 nM ATP detected in the media (3-fold over the levels measured in unstimulated cells, *p* < 0.01). The corresponding levels of eATP detected in the case of IMR-32 and MCF-7 cells were 71 ± 9 nM (15-fold, *p* < 0.01) and 351 ± 29 nM (5-fold, *p* < 0.001) after stimulation with doxorubicin. It has to be noted that these values represent residual eATP levels at the end of 48 h post-stimulation with doxorubicin. These results indicated that doxorubicin-mediated cell death resulted in a concomitant increase in the levels of eATP in the media which remain significantly high up to 48 h post-stimulation with doxorubicin.

### Conditioned Media From Doxorubicin-Treated Cells Promotes Cancer Cell Migration and Invasion

In order to test whether the released ATP measured in the media obtained from dying cells promoted cancer cell metastasis, we used media obtained from HeLa cells treated with 200 nM doxorubicin (Dx) for 24 h as conditioned medium (CM-Dx) in migration and invasion assays. These assays were done in a Boyden chamber wherein cells were grown on top of a transwell insert in serum-free media while the conditioned media was added in the lower chamber. In the case of invasion assay, cells were grown on top of a thin matrigel layer on top of the insert. As control, we also used media obtained from unstimulated HeLa cells (CM-Ctrl). We found that conditioned media obtained from doxorubicin-treated cells (CM-Dx) significantly increased both migration and invasion in all three cell-lines (Figure 1B). CM-Dx-mediated 1.8-fold and 9-fold increase in migration and invasion, respectively, in HeLa cells (*p* < 0.001). Similarly, in MCF-7 cells, CM-Dx-mediated 1.5-fold and 10-fold increase in migration and invasion, respectively (*p* < 0.001). The highest migration (3-fold) and invasion (80-fold) was observed in the case of IMR-32 cells (*p* < 0.001). To confirm that the ATP present in the conditioned media was acting through P2 receptors, we co-stimulated the cells with suramin, a pan-P2 receptor antagonist. In all the three cell-lines, suramin significantly blocked both migration and invasion induced by CM-Dx (*p* < 0.001). We also confirmed that the presence of doxorubicin in CM-Dx did not influence cell migration by stimulating cells directly with doxorubicin. We found no significant change in the number of migrating cells when treated with doxorubicin alone (Figure 1B). A recent report shows that doxorubicin mediates MCF-7 cell migration through rhoA/myosin light chain 2 pathway (Liu et al., 2019). However, in that report cells were treated with doxorubicin for 3 h in a 6-well plate followed by their culture in the Transwell insert where migration was checked after 48 h. In our experiment, cells were directly stimulated on the Transwell insert and migration was checked after 12 h in the case of MCF-7 cells. Overall, our results suggest that media obtained from dying cells contains ATP whose effect on tumor cell migration and invasion could be blocked by a non-specific P2 receptor antagonist, suramin.

### Exogenous ATP Mediates Migration in Different Cancer Cells

Since suramin significantly inhibited CM-Dx-mediated cell migration and invasion, we further confirmed the role of purinergic receptors in this process by directly adding ATP to the media exogenously. To test the concentration of ATP that promotes significant migration in cancer cells, we exogenously added ATP at different doses and measured cell migration using the Boyden chamber assay (Figure 1C). We found a dose-dependent increase in cell migration only in the case of HeLa cells which showed significant numbers of migrated cells from 10 µM onwards with the highest seen with 1 mM ATP. In the case of IMR-32 cells, maximal migration was observed with 30 µM ATP (*p* < 0.001) and was insignificant at 1 mM ATP. MCF-7 cells, on the other hand, showed significant migration at 100 µM (*p* < 0.001). Based on these preliminary observations, ATP was used at 1 mM for HeLa cells, 30 μM for IMR-32 cells and 100 μM for MCF-7 cells in all the subsequent experiments.

In order to further confirm that ATP is responsible for the migration of cells in the Boyden chamber assay, we used an ATP degrading enzyme, apyrase, which hydrolyzes ATP to AMP and inorganic phosphate. Exogenously added ATP increased migration in HeLa cells by 4.7-fold which was significantly inhibited in wells which were also treated with apyrase (Figure 1D). Similarly, the increase in eATP-mediated migration in IMR-32 and MCF-7 cells was also significantly inhibited by 45% in cells that were also co-treated with apyrase (*p* < 0.001 with respect to ATP-treated cells). These results suggest that migration is mediated through ATP, and that its hydrolysis by apyrase abolishes this property in all the three cell-lines.

### Suramin Inhibits Cell Migration and Invasion Mediated by Exogenous ATP

The inhibition of CM-Dx-mediated cell migration by suramin and eATP-mediated migration by apyrase suggest that eATP acts through purinergic receptors. Accordingly, the effect of pan-P2 receptor antagonist, suramin, was tested in all three cell-lines. In the Boyden chamber assay, the number of migrated cells was significantly reduced by 77% in the case of HeLa cells which were treated with both ATP and suramin (Figure 1D). In the same way, we found 70 and 64% reduction in the number of migrated cells in the case of IMR-32 and MCF-7 cells which were co-treated with suramin (*p* < 0.001 with respect to ATP-treated cells). This suggested that eATP-mediated migration of cancer cells could be significantly inhibited through the use of a P2 receptor antagonist.

To further confirm that suramin prevented cancer cell progression, migration and proliferation of cells was measured through a wound healing assay wherein a wound/scratch was created in the center of the monolayer (Figures 1E,F). ATP significantly increased the closure of the wound in all three cell-lines (*p* < 0.05). In HeLa cells, the recovered area was 34 ± 2% at 27 h in control cells which was further increased to 44 ± 2% in cells treated with ATP (*p* < 0.001). In contrast, in cells that were co-stimulated with both ATP and suramin, the recovered wound area was the same as in control cells at 35 ± 2%, indicating that suramin blocked ATP-mediated wound closure (*p* < 0.01 with respect to ATP-treated cells). Similarly, in IMR-32 cells, 76 ± 3% wound was recovered in control cells at 18 h, while in ATP-treated cells the recovered wound was 89 ± 2% (*p* < 0.001). As in the case of HeLa cells, treatment with suramin failed the recovery of the wound and remained at 77 ± 5% (*p* < 0.05). In the case of more proliferative MCF-7 cells, the scratch was recovered by 35 ± 2% within 12 h in control cells which was greatly enhanced in cells treated with ATP (64 ± 1%, *p* < 0.001). In cells that were treated with both ATP and suramin, only 53 ± 1% area was recovered (*p* < 0.05 compared to ATP-treated cells). These results indicated that exogenous ATP mediated accelerated wound closure which was significantly inhibited through the use of a pan-P2 receptor antagonist.

Supporting this data, we also found that eATP mediated 7-fold increase in HeLa cells invasion through the matrigel (*p* < 0.001) which was also significantly inhibited by suramin (Figure 1G). In the case of IMR-32 cells, the invasion process was strongly inhibited with only 43% cells invaded compared to cells treated with eATP (*p* < 0.001). Similar result was observed with MCF-7 cells, wherein eATP-mediated invasion could be significantly inhibited by suramin. These results support the role of P2 receptors in cell migration and invasion.

### P2X7 Receptor Antagonists Inhibit eATP-Mediated Migration of IMR-32 and MCF-7 Cells

Since suramin could significantly inhibit eATP-mediated migration and invasion in all the three cell lines, we screened the expression of eight P2 receptors (3 P2X and 5 P2Y) through real time PCR. Among the ionotropic receptors, we found strong expression of P2X4 receptors in MCF-7 cells while P2X7 receptors were predominant in the IMR-32 cells (Table 2). Among the G protein coupled receptors, we found P2Y6 receptors to be strongly expressed in both HeLa and MCF-7 cells.

**TABLE 2:** Relative expression of P2 receptors in the three cell-lines.

	HeLa^a^	IMR-32	MCF-7
P2X1	21.6	17.9	18.7
P2X4	14.2	17.4	**9.7**
P2X7	17.7	**12.9**	15.2
P2Y1	22.4	n.d.	15.3
P2Y2	13.8	17.1	13.6
P2Y4	17.1	14.7	17.5
P2Y6	**7.9**	16.0	**11.8**
P2Y12	15.8	16.7	14.6

^a^ Total mRNA was isolated from unstimulated HeLa, IMR-32 and MCF-7 cells and cDNA synthesized.

Real time PCR analysis was done using specific primers given in Table 1. The mean δC_t_ values obtained from cDNA synthesized from at least three different RNA preparations is depicted here. The δC_t_ value was calculated from the C_t_ values of the receptor with that of the C_t_ value for β-actin. The mean C_t_ value for β-actin was 12.5 ± 0.2 for HeLa cells, 17.6 ± 0.5 for IMR-32 cells, and 13 ± 0.1 for MCF-7 cells. Entries in bold are receptors with highest expression based on dCt method. n.d.: not determined

**FIGURE 2:**
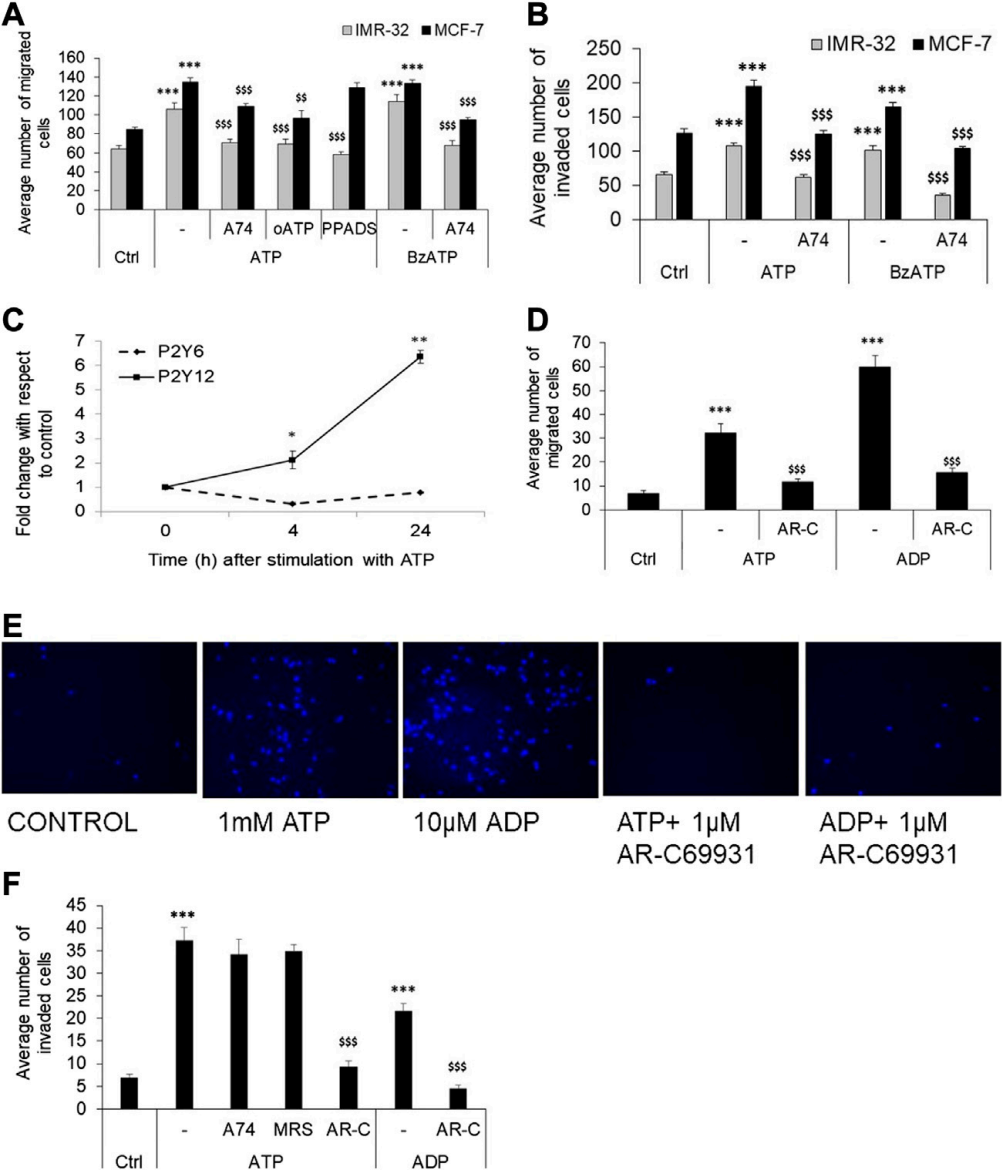
P2X7 and P2Y12 receptor antagonists abolish cancer cell migration and invasion. **(A)** IMR-32 (gray bars) or MCF-7 (black bars) cells were plated on top of a transwell insert and treated with ATP (30 μM for IMR-32 or 100 μM for MCF-7), or 1 μM BzATP, in the presence or absence of 1 μM A740003 (A74), 1 μM oxidized ATP (oATP), or 1 μM PPADS. The number of migrated cells were stained, counted and depicted as mean ± SEM. Student’s *t*-test shows significance of *p* < 0.001 with respect to unstimulated (***) or ATP/BzATP-treated (^$$$^) cells (*n* = 5 independent experiments). **(B)** Invasion assay as described in Methods was done in IMR-32 (gray bars) or MCF-7 (black bars) cells stimulated with ATP (30 μM for IMR-32 or 100 μM for MCF-7) or 1 μM BzATP, in the presence or absence of 1 μM A740003 (A74). The number of cells invading through the matrigel were counted and depicted as mean ± SEM. Student’s *t*-test shows significance of *p* < 0.001 with respect to unstimulated (***) or ATP/BzATP-treated (^$$$^) cells. **(C)** HeLa cells were treated with 1 mM ATP, followed by RNA isolation at 4 and 24 h post-stimulation, for the analysis of P2Y6 and P2Y12 receptors. Fold change was calculated using δδC_t_ method. Significances were calculated with respect to control cells (*p < 0.05, or **p < 0.01) using paired *t*-test. (*n* = 5 independent experiments) **(D)** HeLa cells were grown in the transwell chambers and stimulated with ATP (1 mM), or ADP (10 μM), with or without AR-C 69931 (1 μM, AR-C). The number of cells migrated to the lower chamber after 24 h was counted and represented as mean ± SEM. Student’s *t*-test was used to calculate significance with respect to control cells (***p < 0.001), or with respect to ATP or ADP in the case of AR-C 69931 (^$$$^ *p* < 0.001). **(E)** Representative images of HeLa cells, stained with Hoechst 44,432, post-migration in the transwell assay is depicted here. **(F)** HeLa cells were grown in the transwell chambers on top of a matrigel layer and stimulated with ATP (1 mM), or ADP (10 μM), with or without 1 μM AR-C 69931 (AR-C), 1 μM MRS 2500 (MRS), or 1 μM A740003 (A74). Data is depicted as given for **(D)**.

Further supporting these results, we found that exogenous stimulation of IMR-32 and MCF-7 cells with ATP enhanced P2X7 receptor expression by 2 ± 0.25-fold (*p* < 0.01), an effect also observed with the P2X7 receptor agonist, BzATP. Accordingly, we checked the effect of P2X7 receptor stimulation on cell migration and invasion. In both IMR-32 and MCF-7 cells, BzATP significantly increased cell migration, similar to the effect seen with eATP (Figure 2A). Furthermore, both eATP- and BzATP-mediated cell migration could be significantly inhibited by the P2X7 receptor-specific antagonist, A740003 (*p* < 0.001). A740003 has an IC_50_ of 40 nM for P2X7 receptors and >100 µM for all other P2X receptors (Illes et al., 2020), suggesting that eATP-mediated migration is dependent on P2X7 receptors. This was further confirmed through the use of another P2X7 receptor antagonist, oxidized ATP (oATP), which also showed significant inhibition by approximately 30%. Although BzATP has a higher specificity to P2X7 receptors (EC_50_ of 5 µM) compared to ATP itself (EC_50_ 100 µM), it also shows higher affinity to P2X1, P2X2, and P2X3 receptors (EC_50_ 2 nM for P2X1 receptors) (Illes et al., 2020). Accordingly, we used a non-selective antagonist of P2X receptors, PPADS, which has an EC_50_ of 1 µM for P2X1, P2X2, and P2X3 receptors and 10–50 µM for P2X7 receptors (>500 µM for P2X4 receptors). PPADS (used at 1 μM here) showed 40% inhibition of eATP-mediated migration in the case of IMR-32 cells but had no effect on MCF-7 cells. This may likely suggest that other P2X receptors may be involved in eATP-mediated migration in IMR-32 cells; however, the significant inhibition by at least two specific antagonists of P2X7 receptors—A740003 and oATP—in both IMR-32 and MCF-7 cells points out to their role in metastasis.

Confirming these results, the invasion assays also showed that both ATP and BzATP mediated significant increase in the number of cells invading through the matrigel, which could be inhibited in wells where A740003 was used (Figure 2B). These results indicate that P2X7 receptors might play an important role in migration and invasion properties of IMR-32 and MCF-7 cells.

### eATP-Mediated Migration in HeLa Cells Is P2Y12 Receptor-dependent

Although P2Y6 receptors were strongly expressed in HeLa cells, there was no change in their expression levels when stimulated with eATP (Figure 2C). In contrast, we found a potent increase in the expression of P2Y12 receptors by 2 ± 0.3-fold within 4 h of stimulation, and 6 ± 0.2-fold increase 24 h post-stimulation (*p* < 0.001). In order to identify the role of these receptors in HeLa cell migration and invasion, we used P2Y12 receptor agonist, ADP, and P2Y12 receptor antagonist, AR-C 69931, in the transwell assays. ADP has a specificity for P2Y1 (EC_50_ 5.09 µM), P2Y12 (EC_50_ 7.22 µM) and P2Y13 (EC_50_ 7.94 µM) receptors, while AR-C 69931 acts as a competitive antagonist at P2Y12 receptors (IC_50_ 9.4 µM) (Jacobson et al., 2020). A 4.7-fold increase in the migration of HeLa cells was observed with 1 mM ATP which was reduced by 65% in wells co-stimulated with AR-C 69931 (*p* < 0.001, Figures 2D,E). Furthermore, 10 μM ADP mediated a 9-fold increase in migration (60 ± 5 cells migrated in comparison to 32 ± 4 in ATP-stimulated condition and 7 ± 1 in case of control), which was also significantly inhibited by AR-C 69931 (*p* < 0.001).

In the invasion assay, 1 mM ATP mediated a 5-fold increase in invasion which was reduced by 75% when co-stimulated with AR-C 69931 (*p* < 0.001) (Figure 2F). To rule out the possible role of P2Y1 receptors, we used a very potent and selective antagonist of P2Y1 receptor, MRS2500 (EC_50_ 9.02 µM). There was no inhibition of ATP-mediated invasion in cells co-treated with MRS2500, suggesting that P2Y1 receptors may not be involved in this process, whose expression was also weak in HeLa cells (Table 2). Furthermore, since the dose of ATP used was very high, the likely involvement of P2X7 receptors was also ruled out through the use of the specific antagonist, A740003, which showed no inhibition of ATP-mediated invasion. Therefore, among the various inhibitors tested here, we found P2Y12 antagonist to be the most potent in inhibiting ATP-mediated invasion in HeLa cells. Accordingly, we found a 3-fold, but significant, increase in the number of invading cells with 10 μM ADP (*p* < 0.001), which was also reduced by 79% when co-stimulated with AR-C 69931. These results suggest that in HeLa cells P2Y12 receptors may play a major role in cell migration and invasion.

### eATP-dependent Migration and Invasion of Tumor Cells Is Abolished by COX-2 Inhibitors

**FIGURE 3:**
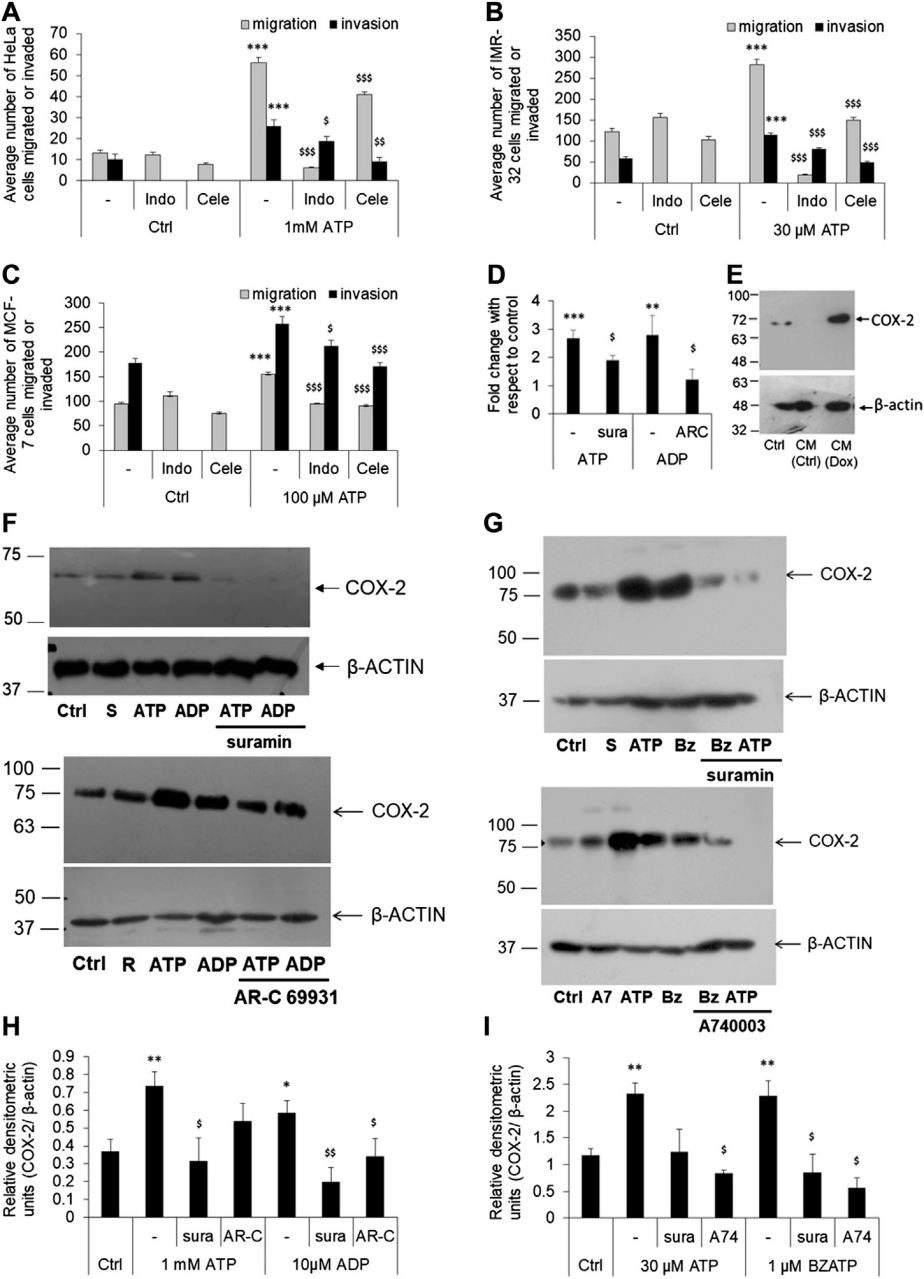
P2 receptor-mediated cell migration or invasion is dependent on COX-2 expression. **(A–C)** HeLa **(A)**, IMR-32 **(B)** or MCF-7 **(C)** cells were plated on transwell inserts directly (for migration assay) or on top of a layer of matrigel (for invasion assays) and stimulated with ATP, with or without indomethacin (indo), or celecoxib (Cele)—both used at 100 nM. After 24 h, migrated/invaded cells from the bottom of the chamber were stained, counted, and depicted here as average number of cells ±SEM. Significances were calculated with respect to control cells (***p < 0.001) for ATP-treated cells, or with respect to ATP for the antagonists (^$^ *p* < 0.05, ^$$^ *p* < 0.01, ^$$$^ *p* < 0.001) (*n* = 5 independent experiments). **(D)** HeLa cells were stimulated with ATP (1 mM), alone or with suramin (sura, 10 μM), or with ADP (10 μM), alone or with AR-C 69931 (AR-C, 1 μM). Total RNA was isolated for cDNA synthesis followed by real time PCR analysis for COX-2 expression. Fold change was calculated using δδC_t_ method using β-actin as a housekeeping control. Significances were calculated with respect to control cells (**p < 0.01, or ***p < 0.001) for the agonists, or with respect to ATP or ADP in the case of antagonists (^$^ *p* < 0.05) through paired *t*-test. **(E)** HeLa cells were treated with conditioned media (CM) obtained from doxorubicin-treated (CM-Dox) or normal (CM-Ctrl) cells as described in Figure 1B. 24 h after stimulation cell lysates were prepared for COX-2 expression. **(F,H)** HeLa cells were stimulated with 1 mM ATP or 10 μM ADP, with or without 10 μM suramin (sura) or 1 μM AR-C 69931 (AR-C) for 24 h. Cell lysates were then collected and run on SDS-PAGE for analysis of COX-2 expression. β-actin was used as a housekeeping control for relative densitometric analysis. A representative Western blot image is shown in F (S, suramin; R, AR-C 69931). Replicates of four Western blots from different passages of cells were used for densitometric analysis. **(G,I)** IMR-32 cells were stimulated with 30 μM ATP or 1 μM BzATP, with or without 10 μM suramin (sura) or 1 μM A740003 (A74) for 24 h. A representative image is shown in F (S, suramin; A7, A740003; Bz, BzATP) and densitometric analysis in **(I)**. Significances were calculated with respect to control cells (*p < 0.05, **p < 0.01) for the agonists, or with respect to ATP or ADP/BzATP in the case of antagonists (^$^ *p* < 0.05, ^$$^ *p* < 0.01).

We had previously hypothesized that COX-2 was a downstream enzyme whose expression is modulated following the activation of the P2 receptor pathway (Fiebich et al., 2014). Therefore, we first wanted to identify whether COX-2 was required for P2 receptor-dependent migration and invasion of tumor cells. We used indomethacin, a nonselective COX inhibitor (IC_50_ 230 nM for COX-1 and IC_50_ 630 nM for COX-2), and celecoxib, a COX-2 specific inhibitor (IC_50_ 15 µM for COX-1 and 40 nM for COX-2). The inhibitors alone had no effect on cell migration in all the three cell-lines (Figures 3A–C). Addition of ATP led to 4-fold increase in migration and 2.5-fold increase in invasion in HeLa cells. However, co-stimulation with indomethacin significantly reduced both migration and invasion by 89% (*p* < 0.001) and 27% (*p* < 0.05). Celecoxib, on the other hand, reduced migration and invasion by 27% (*p* < 0.001) and 64% (*p* < 0.01).

Similar observations were made in the case of IMR-32 and MCF-7 cells too. In IMR-32 cells, eATP mediated a 2-fold increase in migration (*p* < 0.001, Figure 3B) which was reduced by 94% by indomethacin and 47% by celecoxib (both, *p* < 0.001). In the same manner, eATP-mediated invasion in IMR-32 cells was also reduced by 30% by indomethacin and by 58% by celecoxib (*p* < 0.001). In the case of MCF-7 cells, indomethacin inhibited eATP-mediated migration by 39% (*p* < 0.001) and invasion by 18% (*p* < 0.05) while celecoxib inhibited migration by 42% and invasion by 34% (both *p* < 0.001, Figure 3C). These results suggested that, across all the three cell-lines tested, exogenously added ATP mediated cancer cell migration or invasion required the activity of the enzyme COX-2.

### eATP Mediates COX-2 Expression in Cancer Cell-Lines

Since inhibitors of COX-2 abolished P2 receptor-mediated migration and invasion in cancer cell-lines, we wanted to know whether exogenously added ATP affected COX-2 synthesis. In HeLa cells, stimulation with 1 mM ATP increased COX-2 mRNA expression by 2.6 ± 0.3-fold, which was significantly inhibited by suramin (*p* < 0.05, Figure 3D). A similar increase in COX-2 expression was also observed with 10 μM ADP, which was strongly inhibited by AR-C 69931. We then looked at the expression of COX-2 at the protein level for which we first tested the conditioned media obtained from dying cells. Conditioned media (CM) obtained from untreated cells (CM-Ctrl), did not show any COX-2 expression while cells that were exposed to CM obtained from doxorubicin-treated cells (CM-Dox) showed a strong increase in COX-2 expression (Figure 3E), suggesting that CM-Dox contains sufficient ATP coming from the dead cells to induce COX-2 expression in naïve cells.

Changes in the COX-2 mRNA were also reflected at the protein level. We found a significant increase in COX-2 expression in HeLa cells stimulated with either ATP (at 1 mM) or ADP (at 10 μM, Figures 3F,H). Furthermore, P2 receptor antagonists abolished this induced COX-2 expression. In the HeLa cells, the pan-P2 receptor antagonist suramin and the P2Y12 receptor-specific AR-C 69931 significantly inhibited COX-2 synthesis mediated by ATP or ADP. Similarly, in IMR-32 cells, there was a 2-fold increase in the levels of COX-2 in cells stimulated with 30 μM ATP or 1 μM BzATP (*p* < 0.01, Figures 3G,I). This increase in COX-2 expression was significantly reduced by the P2X7 receptor antagonist, A740003 (*p* < 0.01). These results indicated that eATP-mediated activation of P2Y12 receptors in HeLa cells or P2X7 receptors in IMR-32 cells show a common downstream target resulting in increased COX-2 expression in these cells.

### P2 Receptor Activation Enhances MMP-2 Activity

**FIGURE 4:**
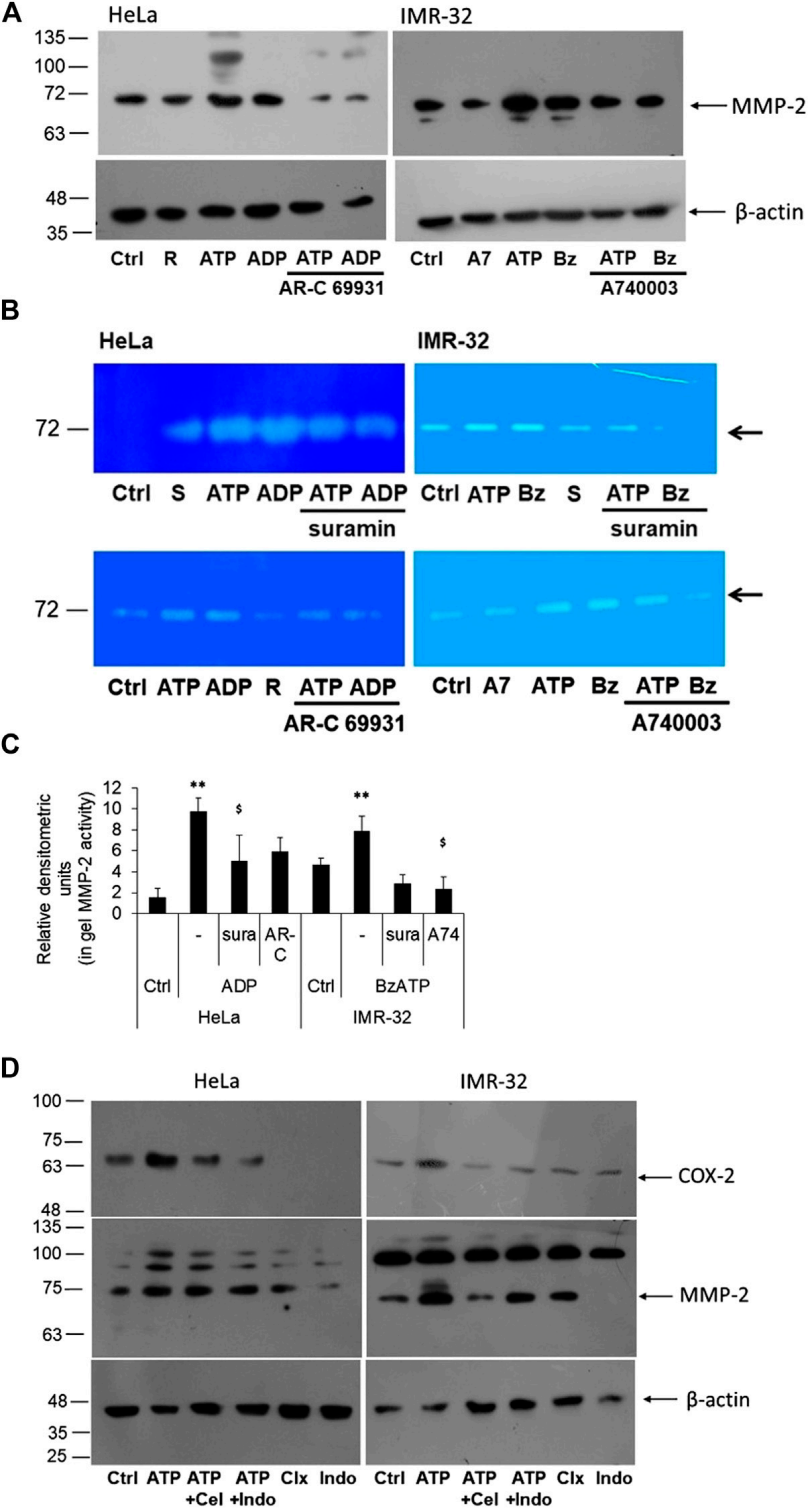
P2 receptor antagonists block eATP-mediated MMP-2 activity. **(A)** HeLa and IMR-32 cells were stimulated with ATP (1 mM or 30 μM, respectively), 10 μM ADP or 1 μM BzATP (Bz), with or without the inhibitors AR-C 69931 (R, 1 μM) or A740003 (A7, 1 μM). 24 h post-stimulation cell lysates were prepared for the detection of MMP-2 through Western blotting as described in Materials and Methods. β-actin was used as a housekeeping control. **(B)** MMP-2 activity was observed through an in-gel assay as described in the Materials and Methods with cells stimulated in the same manner as described in **(A)** and also including the inhibitor suramin (S, 10 μM). Representative zymograph is depicted here. **(C)** Relative densitometry was made based on three independent gelatin zymography experiments. Student’s *t*-test was done to calculate significance of agonist-treated cells with their unstimulated counterparts (**p < 0.01) and the antagonists with respect to their agonist counterparts (^$^ *p* < 0.05). **(D)** HeLa and IMR-32 cells were stimulated with ATP (1 mM for HeLa and 30 μM for IMR-32 cells) for 24 h, with or without celecoxib (100 nM) or indomethacin (100 nM). Cell lysates were probed for the detection of COX-2 or MMP-2 levels with β-actin used as a housekeeping control.

An important enzyme required for the invasiveness of cancer cells is gelatinase or matrix metalloproteinases, of which matrix metalloproteinase 2 (MMP-2) has been widely reported in various cell culture studies (Hsu et al., 2015; Roomi et al., 2017). Therefore, we wanted to know whether exogenous addition of ATP also increased MMP-2 expression in these cells using Western blot and gelatin zymography. There was a moderate increase in the levels of intracellular MMP-2 in both HeLa and IMR-32 cells which could be inhibited through AR-C 69931 and A740003 in HeLa and IMR-32 cells, respectively (Figure 4A). Since MMP-2 is a secreted protein, we collected the media supernatant from stimulated cells and checked for its functional activity through in-gel gelatinase activity. In both HeLa and IMR-32 cells, eATP treatment showed marked increase in gelatinase activity which could be seen reduced in cells which were co-treated with suramin (Figures 4B,C). The P2Y12 receptor agonist ADP showed 6-fold increase in MMP-2 activity in HeLa cells (*p* = 0.01), which could be significantly reduced in cells which were also treated with suramin by 49% (*p* < 0.05). Similarly, in IMR-32 cells, the P2X7 receptor agonist BzATP (1 μM)-mediated increase in MMP-2 activity could be significantly reduced with its specific antagonist A740003 by 70% (*p* < 0.05). These results demonstrate that P2 receptor activation increases the activity of MMP-2, an important metastatic marker responsible for cell invasion.

In order to find out if the activity of MMP-2 was dependent on COX-2 expression, we stimulated the cells with celecoxib and indomethacin, 30 min prior to stimulation with ATP. We first confirmed the effect of the inhibitors on COX-2 levels. We found that both celecoxib and indomethacin reduced ATP-mediated increase in COX-2 levels in both HeLa and IMR-32 cells (Figure 4D). This suggested that these inhibitors not only inhibit the activity of the enzyme but also its expression within the cells. However, the effect of these inhibitors on MMP-2 expression was not remarkable, though in IMR-32 cells, celecoxib showed reduction in the active form of MMP-2.

### P2 Receptor Activation Upregulates p42/44 and p38 MAPK Activity

**FIGURE 5:**
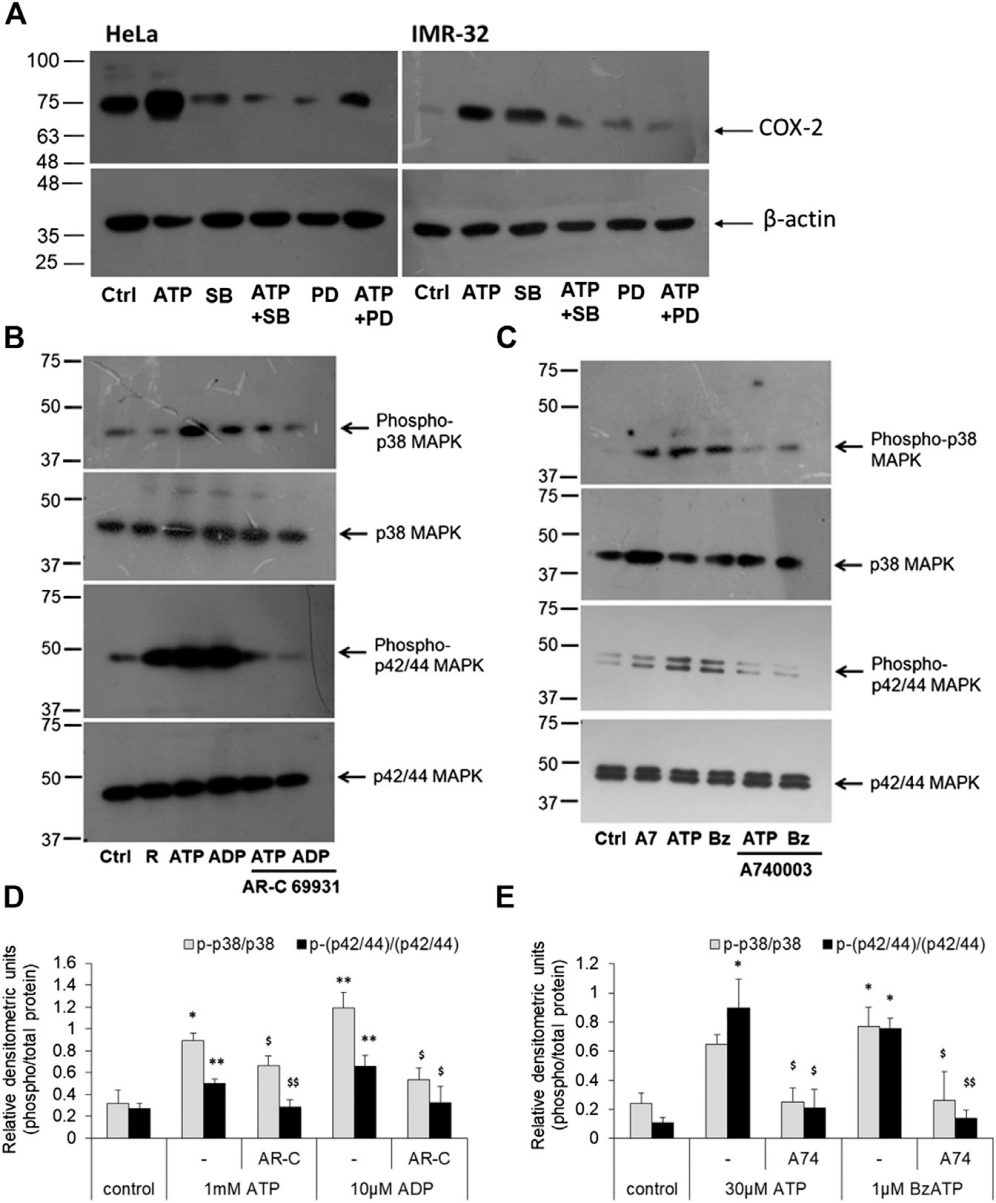
P2 receptor stimulation increases p42/44 MAPK and p38 MAPK activity. **(A)** HeLa or IMR-32 cells were stimulated with ATP (1 mM for HeLa and 30 μM for IMR-32 cells) in the presence or absence of SB202190 (1 μM) or PD98059 (1 μM). The inhibitors were added 30 min prior to the addition of ATP and cells were harvested after 24 h for the detection of COX-2 protein. **(B)** HeLa cells were stimulated with 1 mM ATP or 10 μM ADP, with or without 1 μM AR-C 69931 (R) for 30 min. Cell lysates were then separated on SDS-PAGE and analyzed for phosphorylated or total forms of p42/44 MAPK or phosphorylated or total p38 MAPK. **(C)** IMR-32 cells were stimulated with 30 μM ATP or 1 μM BzATP (Bz), with or without 1 μM A740003 (A7), for 30 min. Cell lysates were then analyzed as described for HeLa cells. Representative images of Western blot are shown from a total of five independent experiments. **(D,E)** Densitometric analysis of phosphorylated p38 or p42/44 MAPK was done with respect to total p38 or p42/44 MAPK, respectively, for HeLa **(D)** or IMR-32 **(E)** cells. Significances were calculated with respect to control cells (*p < 0.05, **p < 0.01), or with corresponding agonists (^$^ *p* < 0.05, ^$$^ *p* < 0.01).

We next investigated the signaling intermediates downstream of P2 receptor activation which were responsible for COX-2 expression. For this we looked at some of the kinases that were known to be involved in the induction of *COX-2* gene. The p42/44 and p38 mitogen-activated protein kinases (MAPK) are major modulators of COX-2 protein in various cells (Parvathenani et al., 2003; Yang et al., 2004; Akundi et al., 2005; Akter et al., 2021). To confirm their involvement, we used two well-known selective and cell-permeant antagonists of these kinases—SB202190, against p38 MAPK (K_d_ 38 nM), and PD98019, against mitogen-activated protein kinase kinase (MKK/MEK; IC_50_ 2–7 µM), which is upstream in the MAPK signaling pathway. We found that in both HeLa and IMR-32 cells, ATP-mediated increase in COX-2 levels could be reduced in cells which were pre-treated with SB202190 or PD98059 (Figure 5A). These results suggested that ATP-mediated increase in COX-2 levels in cancer cells involved the activation of MAPK signaling pathway.

Accordingly, we looked at the activity of MAPKs through phospho-specific antibodies wherein the phosphorylated forms indicate active MAPK form. ATP and the P2Y12 receptor agonist, ADP, significantly increased phosphorylated levels of p42/44 MAPK in HeLa cells by 2-fold (Figures 5B,D). However, the P2Y12 receptor antagonist, AR-C 69931 reduced the phosphorylated form of p42/44 MAPK by 45% in both ATP and ADP-stimulated cells. Similar observation was made in the IMR-32 cells where 30 μM ATP or 1 μM BzATP mediated a significant increase in the activation of p42/44 MAPK (*p < 0.05, Figures 5C,E) which could be abolished by 80% in cells which were pre-treated with the P2X7 receptor antagonist, A740003. These results implicate the role of p42/44 MAPK downstream of the P2 receptor pathway leading to COX-2 synthesis.

A similar effect was seen in the case of p38 MAPK activity. The addition of eATP (1 mM) showed a 3-fold increase in p38 MAPK activity which could be significantly abolished by 26% in cells pre-treated with AR-C 69931 (*p < 0.05, Figures 5B,D). In much the same way, ADP (10 μM) showed 3.7-fold increase in p38 MAPK activity (phosphorylation) which was reduced by 55% in cells treated with P2Y12 receptor antagonist, AR-C 69931. In IMR-32 cells, a 3-fold increase in the phosphorylated levels of p38 MAPK was seen with both ATP (30 μM) and the P2X7 receptor agonist, BzATP (1 μM)(*p < 0.05, Figures 5C,E). Irrespective of the stimulant used, the P2X7 receptor antagonist, A740003, strongly reduced the levels of phosphorylated p38 MAPK by over 60%. These results show that the p38 MAPK are activated downstream of P2X7 receptors in IMR-32 cells and P2Y12 receptors in HeLa cells.

## Discussion

We have hypothesized that eATP, which is found at very high concentrations in the tumor microenvironment, is responsible for the increased expression of COX-2 which in turn affords the cells with migratory and invasive properties turning the tumor into a metastatic one. In order to test this hypothesis, we used three cell-lines of different tissue origins—HeLa (cervical), IMR-32 (neuronal) and MCF-7 (breast). While HeLa and MCF-7 are frequently used in cancer research, IMR-32 was also included in this study because of it being a neuroblastoma derived from a metastatic site in the abdomen and carrying two types of cells—a predominant smaller neuroblast-like cells and larger hyaline fibroblast cells (Tumilowicz et al., 1970). By using three different cell-lines, we wanted to check whether the mechanism of eATP-mediated tumor cell migration and invasion were common across cancer types. Although each of these cell-lines expressed different purinergic receptors, we found that the downstream effector was COX-2 in all cases which affords the cells with enhanced migration or invasion properties.

It has been well-established in literature that the levels of ATP increase in the tumor microenvironment (Idzko et al., 2014; Di Virgilio et al., 2018). This is especially true in the case of cancers that are therapeutically targeted wherein death of drug- or radiation-sensitive cells results in changing the contents of the microenvironment, of which ATP, among others, is a prominent molecule (Martins et al., 2009; Schneider et al., 2015; Kloss et al., 2019). We observed that the treatment with doxorubicin also showed an increase in the levels of eATP in the media 48 h post-stimulation. This “residual ATP” is the amount of ATP left in the media after it had been degraded by ectonucleotidases and/or internalized through macropinocytosis besides the ATP that has bound to various P2 receptors. Treatment of acute myeloid leukemia cells with another chemotherapeutic agent, daunorubicin (200 nM), similarly showed the release of ATP in the range of 2–8 nM, 6 h post-stimulation (Pegoraro et al., 2020). The tumor microenvironment, therefore, remains enriched with ATP in the aftermath of chemotherapy. The amount of residual ATP depends on various factors such as the intracellular levels of ATP, which in turn depends on the metabolic activity of the cell, percentage of cells dying in response to the chemotherapeutic agent, and the expression levels and activity of membrane ectonucleotidases which are responsible for the degradation of eATP. This explains the differences we found in the levels of residual ATP between the three cell types. Among those we tested, HeLa cells were most sensitive to doxorubicin (53% cell death at 48 h) and therefore showed a high level of residual ATP. However, MCF-7 cells, despite slightly lower cell death at 41%, had the most measured ATP levels due to their higher proliferation rate. We have shown that this “residual ATP” has the ability to afford cancer cells with migration, invasion and increased expression of COX-2 through the use of conditioned media. Treatment of the cells with apyrase or suramin abolished eATP-mediated migration of cancer cells suggesting that the residual ATP is directly responsible for cell migration through P2 receptors. In light of such observations, ectonucleotidases such as CD73 and CD39 are increasingly gaining recognition as the new targets in cancer therapeutics (Moesta et al., 2020).

In this study, we screened eight P2 receptors which were commonly reported in cancer—the vast majority of these studies relate to P2X7, P2Y2 and P2Y12 receptors (Adinolfi et al., 2012; Li et al., 2013; Chadet et al., 2014; Jin et al., 2014). P2X7 receptors have been shown to express strongly in prostate cancer cells, in osteosarcoma, in lymphocyte-infiltrating gastric cancer, pancreatic ductal adenocarcinoma cell lines, and in patients with malignant pleural mesothelioma, and its antagonism has been shown to inhibit growth of tumors in various *in vivo* models (Qiu et al., 2014; Giannuzzo et al., 2015; Amoroso et al., 2016; Zhang et al., 2019; Calik et al., 2020). We found this true in the case of IMR-32 neuroblastoma and MCF-7 breast cancer cells. In both these cell types, BzATP significantly increased migration and invasion mediated by these cells, which could be inhibited through the use of P2X7 receptor-specific antagonist, A740003, and oATP. Antagonising P2X7 receptors has been shown to inhibit tumor growth in the case of human gliomas (Kan et al., 2020). In neuroblastoma cells, P2X7 inhibition not only reduced tumor progression but was also shown to influence metabolic activity via Akt pathway and angiogenesis through reduced vascular endothelial growth factor (VEGF) secretion (Amoroso et al., 2015). In the MDA-MB-231 cells, activation of P2X7 receptors induces calcium-activated SK3 potassium channels which assists in cell migration (Jelassi et al., 2011). P2X7 receptor has been shown to induce fast F-actin reorganization and formation of filopodia, thereby promoting invasion (Brisson et al., 2020). These roles of eATP-mediated morphological changes in early metastasis have also been shown to depend not only on P2X7 receptor stimulation but also on eATP which gets internalized through macropinocytosis (Cao et al., 2019). In addition to these roles, P2X7 receptors also modulate the tumor microenvironment wherein blockade of the receptors through systemic administration of A740003 showed an increase in CD4^+^ cells but with diminished expression of ectonucleotidases, CD39 and CD73 (De Marchi et al., 2019). These observations and the current study support the various reports that have championed P2X7 receptors as a good therapeutic target. This is also reflected in the various preclinical studies which use P2X7 receptor antagonists as potential anti-tumour drugs (Di Virgilio et al., 2018; Lara et al., 2020).

We do not rule out the possible role of P2X4 receptors, which are the predominantly expressed receptors in MCF-7 cells, and of P2Y2 receptors in tumor progression. In the relatively more potent and metastatic breast cancer cell-line, MDA-MB-231, and in prostate cancer cells, P2Y2 receptors have been shown to be strongly expressed and whose knockdown prevented cell migration and invasion (Li et al., 2013; Eun et al., 2015). P2Y2 receptors further mediate inflammasome formation in radiotherapy-resistant breast cancer cells enhancing their invasiveness (Jin et al., 2018). MDA-MB-231 cells also strongly express P2Y6 receptors whose blockage with MRS 2578 or downregulation using siRNA inhibited migration and invasion (Ma et al., 2016). In P2Y6 knockout mice, lung tumor metastasis was prevented by reducing the recruitment of neutrophils (Qin et al., 2020). However, in our studies, we saw that there was no change in the levels of P2Y6 mRNA in cells stimulated with ATP unlike P2Y12 mRNA whose expression increased by 6-fold in 24 h. The expression of P2Y6 receptor has instead been shown to increase following hypoxia or epidermal growth factor treatment in MDA-MB-231 cells (Azimi et al., 2016). A specific antagonist of P2Y12 receptors, AR-C 69931, also significantly inhibited both ATP and ADP-mediated cell migration and invasion. P2Y12 receptor antagonists such as clopidogrel and ticagrelor are currently under clinical studies in breast, pancreatic and head and neck cancer (Ballerini et al., 2018; Elaskalani et al., 2020).

The fact that different cell types show upregulation of different P2 receptors suggests that it is essential to target each cancer type with the specific receptor expressed on its surface, in response to its microenvironment. For instance, in the case of hepatocellular carcinoma, P2Y11 receptors have been shown to play a major role in cell migration through ATP-induced calcium signaling (Khalid et al., 2017). P2Y11 receptors have also been implicated in pancreatic cancer cell migration through a p38 MAPK-dependent pathway (Shi et al., 2013). In the case of renal cancer cells, P2X6 receptors were found to increase cell migration and invasion involving calcium-mediated p42/44 MAPK signaling and MMP-9 activation (Gong et al., 2019). The diversity of P2 receptors, their promiscuity to different nucleotides, and the presence of ectonucleotidases, makes it difficult to target any one single receptor for a given cancer type (Fiebich et al., 2014; da Silva Ferreira et al., 2019). Moreover, mere expression of a receptor at high levels may not reflect its increased activity. This is especially true of P2X7 receptors whose opening would increase membrane permeability leading to cell death. However, in the case of tumors, it has been found that there is, instead, an increase in the expression of a non-pore forming P2X7 receptor which is functionally redundant, thereby assisting tumor cell survival (Gilbert et al., 2019). The two splice variants of P2X7 receptors also show differential modulation in response to chemotherapy as seen in acute myeloid leukemia (Pegoraro et al., 2020). It has to also be noted that the physiological role of P2 receptors depends on the cell type they are expressed on—inhibiting P2Y12 receptors with ticagrelor in pancreatic ductal adenocarcinoma cells decreases their proliferative capacity through the Akt pathway (Elaskalani et al., 2020), inhibition of the same receptors on macrophages enhances their tumor cell phagocytic properties involving ER stress pathway (Pavlovic et al., 2020) and inhibits the formation of inflammasome (Huang et al., 2020), whose role in cancer progression continues to be sought (Hamarsheh and Zeiser, 2020). Therefore, targeting specific P2 receptors remains a challenge making therapeutic approach personalized depending on the nucleotide composition of the microenvironment and/or receptor expression. Our study, however, shows that, irrespective of the type of P2 receptor that is activated upstream, the downstream pathway leads to increased COX-2 expression, thereby providing a common pathway for therapeutic intervention. This has been true with both P2X7 and P2Y12 receptors *in vitro*.

COX-2 has been shown to be overexpressed in various cancers (Ristimaki et al., 2002; Yan et al., 2004). In the human lung cancer cells, COX-2 has been shown to afford cells with resistance to apoptosis, thereby making them withstand chemotherapy (Chen et al., 2010). Doxorubicin-resistant MCF-7 cells (MCF-7/DOX), which exhibit high invasiveness, strongly express COX-2 similar to that expressed in the highly metastatic breast cancer MDA-MB-231 cells (Kang et al., 2011). Increased COX-2 activity leads to increased PGE_2_ synthesis, whose levels have been shown to be increased in the tumor microenvironment (Wan et al., 2013; Lala et al., 2018; Carter et al., 2019). Inhibition of COX-2 in MDA-MB-231 cells through upstream inhibition of the nuclear factor κB (NF-κB) signaling suppressed its invasiveness (Kim et al., 2014). The p42/44 MAPK signaling is upstream of the NF-κB-mediated COX-2 expression (Akundi et al., 2005), and has been shown to be activated following P2 receptor stimulation (Parvathenani et al., 2003; Yang et al., 2004; Lili et al., 2019). Similarly, p38 MAPK is essential for COX-2 synthesis, either directly through downstream activation of transcription factors (Fiebich et al., 2000; Akundi et al., 2005), or through stabilization of COX-2 mRNA post-transcription, thereby prolonging the effect of COX-2 or inflammation for a much longer period (Lasa et al., 2000; Harper and Tyson-Capper, 2008). Recently, we found that in macrophages, eATP enhanced LPS-mediated COX-2 synthesis by sustaining high levels of COX-2 mRNA through extended phosphorylation of cyclin-dependent kinase 9 and p38 MAPK (Akter et al., 2021). Our results here show that the presence of residual ATP within the tumor microenvironment provides a low-grade chronic inflammation. The resulting activation of P2 receptors leads to increased phosphorylation of p38 and p42/44 MAPK, thereby leading to increased COX-2 synthesis. Antagonists of P2X7 or P2Y12 receptors, therefore, not only reduce the levels of phosphorylated p38 and p42/44 MAPK, but also show reduction in COX-2 levels and abolish migration/invasive properties of the cancer cells in response to eATP.

Inhibition of COX-2, especially through nonsteroidal anti-inflammatory drugs (NSAIDs), has been considered a therapeutic alternative for arresting tumor growth (Gupta and Dubois, 2001; Ulrich et al., 2006). However, chronic use of non-specific NSAIDs or COX-2 specific inhibitors (COXIBs) for other diseases had been counterproductive (in t’ Veld et al., 2001; Juni et al., 2004). We had previously hypothesized that upstream inhibition of P2 receptors will result in reduced COX-2 expression without affecting its housekeeping role (Fiebich et al., 2014). These P2 receptor antagonists, dubbed as P2 receptor-based anti-inflammatory drugs (PBAIDs), would inhibit the downstream MAPK signaling, COX-2 synthesis and cancer cell migration/invasion. PBAIDs, therefore, provide a suitable alternative to NSAIDs for cancer treatment without the unpleasant gastrointestinal side-effects associated with long-term NSAID usage or the risks associated with COXIBs. PBAIDs such as suramin has been repurposed as an anti-cancer drug in other studies (Ahmed et al., 2016; Cheng et al., 2019; Wiedemar et al., 2020). We have also shown here that suramin inhibits eATP-mediated COX-2 and MMP-2 synthesis and prevents cancer cell migration and invasion. While the new generation specific P2 receptor antagonists have specificity of action on their side, suramin is an already approved drug by US FDA and could be repurposed as a PBAID.

**FIGURE 6:**
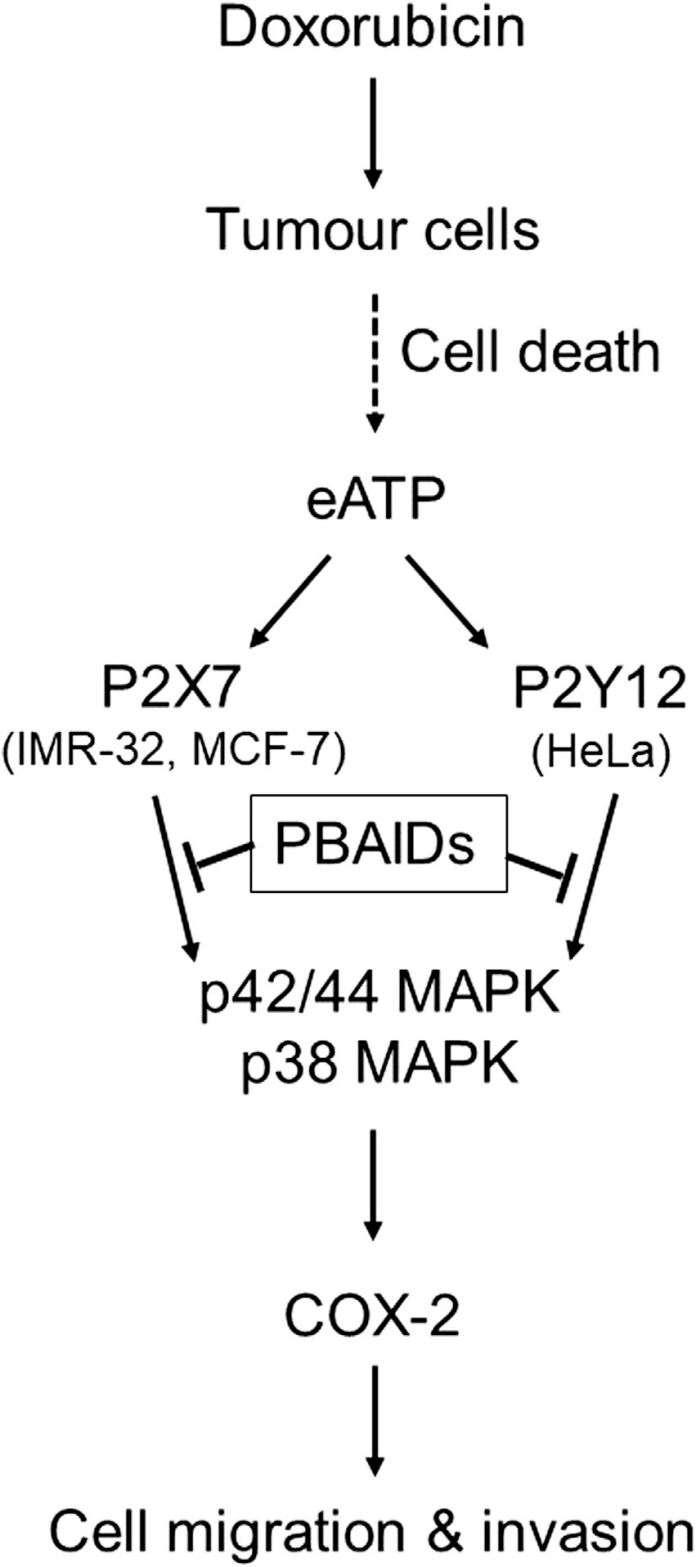
Exogenous ATP mediates increased COX-2 expression leading to cancer cell metastasis. Treatment of tumors with chemotherapeutic agents such as doxorubicin leads to profound cell death and increased levels of exogenous ATP (eATP) in the extracellular milieu. This ATP acts on the P2 receptors of tumor cells in the vicinity through different P2 receptors depending on tumor type such as P2X7 receptors in case of IMR-32 and MCF-7 cells or P2Y12 receptors in case of HeLa cells. Activation of either of these P2 receptors leads to the activation of p38 and p42/44 MAPK which, in turn, induce the transcription of COX-2. Increased COX-2 synthesis has been shown to modulate tumor cell migration and invasiveness, thus contributing toward increased metastasis. P2 receptor-based anti-inflammatory drugs (PBAIDs), which are specific P2 receptor antagonists, have been shown to block the phosphorylation of MAPKs, synthesis of COX-2 and cell migration or invasion.

In summary, we show that doxorubicin mediated cell death leads to an increase in the levels of eATP, which in turn leads to the activation of different P2 receptors depending on the cell type (Figure 6). P2 receptor antagonists are able to inhibit COX-2 expression and MMP-2 activity, thereby confirming that increased levels of eATP are responsible for the metastatic properties of these cells. Blocking of P2 receptors in tumor cells, *in vivo*, leads to reduction in tumor growth due to inhibition of COX-2. This has direct relevance to cancer recurrence where chemotherapeutic agents kill drug-sensitive cells leading to a build-up of eATP in the tumor microenvironment. This ATP may act on the P2 receptors of nearby drug-resistant cells thereby increasing their COX-2 expression. Such cells have the potential to turn metastatic leading to cancer recurrence over a period of time. Therefore, targeting the P2 receptor-COX-2 pathway is a promising strategy in the control of cancer resurgence. The fact that we found different P2 receptors for the different cell lines further suggest that each cancer type has its own specific P2 receptor target and, therefore, earlier reports on P2 receptor antagonists in cancer treatment have to be revisited.

## Data Availability Statement

All the datasets that have been used in the current study are available from the corresponding author upon request.

## Author Contributions

Conceived and planned experiments, RA; experiments performed by SS and HK; manuscript writing and editing, SS and RA.

## Funding

This work was supported in part by grants from the Council of Scientific and Industrial Research (CSIR) [37(1702)/17/EMR-II] and Science and Engineering Research Board (SERB) [EMR/2016/001442] to RA.

## Conflict of Interest

The authors declare that the research was conducted in the absence of any commercial or financial relationships that could be construed as a potential conflict of interest.

## Abbreviations

A740003, N-[1-[[(Cyanoamino)(5-quinolinylamino)methylene]amino]-2,2-dimethyl propyl]-3,4-dimethoxybenzeneacetamide; AR-C 69331, 2-(Propylthio)adenosine-5′-O-(β,γ-difluoromethylene)triphosphate tetrasodium salt; ADP, adenosine diphosphate; ATP, adenosine triphosphate; BzATP, 2′(3′)-O-(4-Benzoylbenzoyl)adenosine-5′-triphosphate tri(triethyl ammonium) salt; COX, cyclooxygenase; eATP, extracellular ATP; MMP, matrix metalloproteinase; MRS2500, 2-iodo-N6-methyl-(N)-methanocarba-2′-deoxyadenosine 3′,5′-bisphosphate; NSAID, nonsteroidal anti-inflammatory drug; oATP, oxidized adenosine triphosphate (adenosine 5′-triphosphate-2′,3′-dialdehyde); PD 98059, 2-(2-amino-3-methoxyphenyl)-4H-1-benzopyran-4-one; PGE2, prostaglandin E2; PPADS, pyridoxalphosphate-6-azophenyl-2′,4′-disulfonic acid; SB 202190, 4-(4-Fluorophenyl)-2-(4-hydroxyphenyl)-5-(4-pyridyl)-1H-imidazole.

## References

[B1] Adinolfi E. Raffaghello L. Giuliani A. L. Cavazzini L. Capece M. Chiozzi P. (2012). Expression of P2X7 receptor increases *in vivo* tumor growth. Cancer Res. 72 (12), 2957–2969. 10.1158/0008-5472.CAN-11-1947 22505653

[B2] Ahmed K. Shaw H. V. Koval A. Katanaev V. L. (2016). A second WNT for old drugs: drug repositioning against WNT-dependent cancers. Cancers 8 (7), 66 10.3390/cancers8070066 PMC4963808 27429001

[B3] Akter S. Sharma R. K. Sharma S. Rastogi S. Fiebich B. L. Akundi R. S. (2021). Exogenous ATP modulates prostaglandin E2 (PGE2) release in macrophages through sustained phosphorylation of CDK9 and p38 MAPK. J. Leukoc. Biol. 10.1002/JLB.3A1219-697RR 33438260

[B4] Akundi R. S. Candelario-Jalil E. Hess S. Hüll M. Lieb K. Gebicke-Haerter P. J. (2005). Signal transduction pathways regulating cyclooxygenase-2 in lipopolysaccharide-activated primary rat microglia. Glia 51 (3), 199–208. 10.1002/glia.20198 15800925

[B5] Amoroso F. Capece M. Rotondo A. Cangelosi D. Ferracin M. Franceschini A. (2015). The P2X7 receptor is a key modulator of the PI3K/GSK3β/VEGF signaling network: evidence in experimental neuroblastoma. Oncogene 34 (41), 5240–5251. 10.1038/onc.2014.444 25619831

[B6] Amoroso F. Salaro E. Falzoni S. Chiozzi P. Giuliani A. L. Cavallesco G. (2016). P2X7 targeting inhibits growth of human mesothelioma. Oncotarget 7 (31), 49664–49676. 10.18632/oncotarget.10430 27391069 PMC5226537

[B7] Avanzato D. Genova T. Fiorio Pla A. Bernardini M. Bianco S. Bussolati B. (2016). Activation of P2X7 and P2Y11 purinergic receptors inhibits migration and normalizes tumor-derived endothelial cells via cAMP signaling. Sci. Rep. 6, 32602 10.1038/srep32602 27586846 PMC5009337

[B8] Avanzini S. Antal T. (2019). Cancer recurrence times from a branching process model. PLoS Comput. Biol. 15 (11), e1007423 10.1371/journal.pcbi.1007423 31751332 PMC6871767

[B9] Azimi I. Beilby H. Davis F. M. Marcial D. L. Kenny P. A. Thompson E. W. (2016). Altered purinergic receptor-Ca²⁺ signaling associated with hypoxia-induced epithelial-mesenchymal transition in breast cancer cells. Mol. Oncol. 10 (1), 166–178. 10.1016/j.molonc.2015.09.006 26433470 PMC5528926

[B10] Ballerini P. Dovizio M. Bruno A. Tacconelli S. Patrignani P. (2018). P2Y12 receptors in tumorigenesis and metastasis. Front. Pharmacol. 9, 66 10.3389/fphar.2018.00066 29456511 PMC5801576

[B11] Bray F. Ferlay J. Soerjomataram I. Siegel R. L. Torre L. A. Jemal A. (2018). Global cancer statistics 2018: GLOBOCAN estimates of incidence and mortality worldwide for 36 cancers in 185 countries. CA: Cancer J. Clin. 68 (6), 394–424. 10.3322/caac.21492 30207593

[B12] Brisson L. Chadet S. Lopez-Charcas O. Jelassi B. Ternant D. Chamouton J. (2020). P2X7 receptor promotes mouse mammary cancer cell invasiveness and tumour progression, and is a target for anticancer treatment. Cancers 12 (9), 2342 10.3390/cancers12092342 PMC7565976 32825056

[B13] Calik I. Calik M. Sarikaya B. Ozercan I. H. Arslan R. Artas G. (2020). P2X7 receptor as an independent prognostic indicator in gastric cancer. Bosn. J. Basic Med. Sci. 20 (2), 188–196. 10.17305/bjbms.2020.4620 32070268 PMC7202194

[B14] Cao Y. Wang X. Li Y. Evers M. Zhang H. Chen X. (2019). Extracellular and macropinocytosis internalized ATP work together to induce epithelial-mesenchymal transition and other early metastatic activities in lung cancer. Cancer Cell Int. 19, 254 10.1186/s12935-019-0973-0 31582910 PMC6771108

[B15] Carter B. Z. Mak P. Y. Wang X. Tao W. Ruvolo V. Mak D. (2019). An ARC-regulated IL1β/Cox-2/PGE2/β-catenin/ARC Circuit controls leukemia-microenvironment interactions and Confers drug resistance in AML. Cancer Res. 79 (6), 1165–1177. 10.1158/0008-5472.CAN-18-0921 30674535 PMC6420856

[B16] Chadet S. Jelassi B. Wannous R. Angoulvant D. Chevalier S. Besson P. (2014). The activation of P2Y2 receptors increases MCF-7 breast cancer cells migration through the MEK-ERK1/2 signalling pathway. Carcinogenesis 35 (6), 1238–1247. 10.1093/carcin/bgt493 24390819

[B17] Chen W. Bai L. Wang X. Xu S. Belinsky S. A. Lin Y. (2010). Acquired activation of the Akt/cyclooxygenase-2/Mcl-1 pathway renders lung cancer cells resistant to apoptosis. Mol. Pharmacol. 77 (3), 416–423. 10.1124/mol.109.061226 19933775 PMC2835422

[B18] Cheng B. Gao F. Maissy E. Xu P. (2019). Repurposing suramin for the treatment of breast cancer lung metastasis with glycol chitosan-based nanoparticles. Acta Biomater. 84, 378–390. 10.1016/j.actbio.2018.12.010 30528604 PMC6362832

[B19] Choi E. M. Heo J. I. Oh J. Y. Kim Y. M. Ha K. S. Kim J. I. (2005). COX-2 regulates p53 activity and inhibits DNA damage-induced apoptosis. Biochem. Biophys. Res. Commun. 328 (4), 1107–1112. 10.1016/j.bbrc.2005.01.072 15707991

[B20] da Silva Ferreira N. C. Alves L. A. Soares-Bezerra R. J. (2019). Potential therapeutic applications of P2 receptor antagonists: from bench to clinical trials. Curr. Drug Targets 20 (9), 919–937. 10.2174/1389450120666190213095923 30760187

[B21] De Marchi E. Orioli E. Pegoraro A. Sangaletti S. Portararo P. Curti A. (2019). The P2X7 receptor modulates immune cells infiltration, ectonucleotidases expression and extracellular ATP levels in the tumor microenvironment. Oncogene 38 (19), 3636–3650. 10.1038/s41388-019-0684-y 30655604 PMC6756114

[B22] Deli T. Csernoch L. (2008). Extracellular ATP and cancer: an overview with special reference to P2 purinergic receptors. Pathol. Oncol. Res. 14 (3), 219–231. 10.1007/s12253-008-9071-7 18575829

[B23] Di Virgilio F. Sarti A. C. Falzoni S. De Marchi E. Adinolfi E. (2018). Extracellular ATP and P2 purinergic signalling in the tumour microenvironment. Nat. Rev. Cancer 18 (10), 601–618. 10.1038/s41568-018-0037-0 30006588

[B24] Elaskalani O. Domenichini A. Abdol Razak N. B. E Dye D. Falasca M. Metharom P. (2020). Antiplatelet drug ticagrelor enhances chemotherapeutic efficacy by targeting the novel P2Y12-AKT pathway in pancreatic cancer cells. Cancers 12 (1), 250 10.3390/cancers12010250 PMC7016832 31968611

[B25] Eun S. Y. Ko Y. S. Park S. W. Chang K. C. Kim H. J. (2015). P2Y2 nucleotide receptor-mediated extracellular signal-regulated kinases and protein kinase C activation induces the invasion of highly metastatic breast cancer cells. Oncol. Rep. 34 (1), 195–202. 10.3892/or.2015.3972 26063340

[B26] Feng D. Zhao T. Yan K. Liang H. Liang J. Zhou Y. (2017). Gonadotropins promote human ovarian cancer cell migration and invasion via a cyclooxygenase 2-dependent pathway. Oncol. Rep. 38 (2), 1091–1098. 10.3892/or.2017.5784 28677781

[B27] Ferrandina G. Lauriola L. Zannoni G. F. Fagotti A. Fanfani F. Legge F. (2002). Increased cyclooxygenase-2 (COX-2) expression is associated with chemotherapy resistance and outcome in ovarian cancer patients. Ann. Oncol. 13 (8), 1205–1211. 10.1093/annonc/mdf207 12181243

[B28] Fiebich B. L. Akter S. Akundi R. S. (2014). The two-hit hypothesis for neuroinflammation: role of exogenous ATP in modulating inflammation in the brain. Front. Cell. Neurosci. 8, 260 10.3389/fncel.2014.00260 25225473 PMC4150257

[B29] Fiebich B. L. Mueksch B. Boehringer M. Hüll M. (2000). Interleukin-1beta induces cyclooxygenase-2 and prostaglandin E(2) synthesis in human neuroblastoma cells: involvement of p38 mitogen-activated protein kinase and nuclear factor-kappaB. J. Neurochem. 75 (5), 2020–2028. 10.1046/j.1471-4159.2000.0752020.x 11032891

[B30] Giannuzzo A. Pedersen S. F. Novak I. (2015). The P2X7 receptor regulates cell survival, migration and invasion of pancreatic ductal adenocarcinoma cells. Mol. Cancer 14, 203 10.1186/s12943-015-0472-4 26607222 PMC4660609

[B31] Gilbert S. M. Oliphant C. J. Hassan S. Peille A. L. Bronsert P. Falzoni S. (2019). ATP in the tumour microenvironment drives expression of nfP2X7, a key mediator of cancer cell survival. Oncogene 38 (2), 194–208. 10.1038/s41388-018-0426-6 30087439 PMC6328436

[B32] Gong D. Zhang J. Chen Y. Xu Y. Ma J. Hu G. (2019). The m6A-suppressed P2RX6 activation promotes renal cancer cells migration and invasion through ATP-induced Ca^2+^ influx modulating ERK1/2 phosphorylation and MMP9 signaling pathway. J. Exp. Clin. Cancer Res. 38 (1), 233 10.1186/s13046-019-1223-y 31159832 PMC6547495

[B33] Gupta R. A. Dubois R. N. (2001). Colorectal cancer prevention and treatment by inhibition of cyclooxygenase-2. Nat. Rev. Cancer 1 (1), 11–21. 10.1038/35094017 11900248

[B34] Hamarsheh S. Zeiser R. (2020). NLRP3 inflammasome activation in cancer: a double-edged sword. Front. Immunol. 11, 1444 10.3389/fimmu.2020.01444 32733479 PMC7360837

[B35] Harper K. A. Tyson-Capper A. J. (2008). Complexity of COX-2 gene regulation. Biochem. Soc. Trans. 36 (Pt 3), 543–545. 10.1042/BST0360543 18482003

[B36] Holleczek B. Stegmaier C. Radosa J. C. Solomayer E. F. Brenner H. (2019). Risk of loco-regional recurrence and distant metastases of patients with invasive breast cancer up to ten years after diagnosis—results from a registry-based study from Germany. BMC Cancer 19 (1), 520 10.1186/s12885-019-5710-5 31146706 PMC6543576

[B37] Houssami N. Macaskill P. Marinovich M. L. Morrow M. (2014). The association of surgical margins and local recurrence in women with early-stage invasive breast cancer treated with breast-conserving therapy: a meta-analysis. Ann. Surg Oncol. 21 (3), 717–730. 10.1245/s10434-014-3480-5 24473640 PMC5705035

[B38] Hsu J. Y. Chang K. Y. Chen S. H. Lee C. T. Chang S. T. Cheng H. C. (2015). Epidermal growth factor-induced cyclooxygenase-2 enhances head and neck squamous cell carcinoma metastasis through fibronectin up-regulation. Oncotarget 6 (3), 1723–1739. 10.18632/oncotarget.2783 25595899 PMC4359327

[B39] Huang B. Qian Y. Xie S. Ye X. Chen H. Chen Z. (2020). Ticagrelor inhibits the NLRP3 inflammasome to protect against inflammatory disease independent of the P2Y12 signaling pathway. Cell. Mol. Immunol. 10.1038/s41423-020-0444-5 PMC8093290 32523112

[B40] Idzko M. Ferrari D. Eltzschig H. K. (2014). Nucleotide signalling during inflammation. Nature 509 (7500), 310–317. 10.1038/nature13085 24828189 PMC4222675

[B41] Illes P. Muller C. E. Jacobson K. A. Grutter T. Nicke A. Fountain S. J. (2020). Update of P2X receptor properties and their pharmacology: IUPHAR Review 30. Br. J. Pharmacol.: 10.1111/bph.15299 PMC8199792 33125712

[B42] in t’ Veld B. A. Ruitenberg A. Hofman A. Launer L. J. van Duijn C. M. Stijnen T. (2001). Nonsteroidal antiinflammatory drugs and the risk of Alzheimer’s disease, N. Engl. J. Med. 345 (21), 1515–1521. 10.1056/NEJMoa010178 11794217

[B43] Jacobson K. A. Delicado E. G. Gachet C. Kennedy C. von Kügelgen I. Li B. (2020). Update of P2Y receptor pharmacology: IUPHAR review 27. Br. J. Pharmacol. 177 (11), 2413–2433. 10.1111/bph.15005 32037507 PMC7205808

[B44] Jelassi B. Chantôme A. Alcaraz-Pérez F. Baroja-Mazo A. Cayuela M. L. Pelegrin P. (2011). P2X(7) receptor activation enhances SK3 channels- and cystein cathepsin-dependent cancer cells invasiveness. Oncogene 30 (18), 2108–2122. 10.1038/onc.2010.593 21242969

[B45] Jiang J. X. Riquelme M. A. Zhou J. Z. (2015). ATP, a double-edged sword in cancer. Oncoscience 2 (8), 673–674. 10.18632/oncoscience.230 26425653 PMC4580055

[B46] Jin H. Eun S. Y. Lee J. S. Park S. W. Lee J. H. Chang K. C. (2014). P2Y2 receptor activation by nucleotides released from highly metastatic breast cancer cells increases tumor growth and invasion via crosstalk with endothelial cells. Breast Cancer Res. 16 (5), R77 10.1186/bcr3694 25156554 PMC4406012

[B47] Jin H. Ko Y. S. Kim H. J. (2018). P2Y2R-mediated inflammasome activation is involved in tumor progression in breast cancer cells and in radiotherapy-resistant breast cancer. Int. J. Oncol. 53 (5), 1953–1966. 10.3892/ijo.2018.4552 30226596 PMC6192788

[B48] Jüni P. Nartey L. Reichenbach S. Sterchi R. Dieppe P. A. Egger M. (2004). Risk of cardiovascular events and rofecoxib: cumulative meta-analysis. Lancet 364 (9450), 2021–2029. 10.1016/S0140-6736(04)17514-4 15582059

[B49] Kan L. K. Seneviratne S. Drummond K. J. Williams D. A. O'Brien T. J. Monif M. (2020). P2X7 receptor antagonism inhibits tumour growth in human high-grade gliomas. Purinergic Signal. 16 (3), 327–336. 10.1007/s11302-020-09705-2 32583309 PMC7524927

[B50] Kang J. H. Song K. H. Jeong K. C. Kim S. Choi C. Lee C. H. (2011). Involvement of Cox-2 in the metastatic potential of chemotherapy-resistant breast cancer cells. BMC Cancer 11, 334 10.1186/1471-2407-11-334 21813027 PMC3199868

[B51] Karavitis J. Zhang M. (2013). COX2 regulation of breast cancer bone metastasis. Oncoimmunology 2 (3), e23129 10.4161/onci.23129 23802065 PMC3661150

[B52] Khakh B. S. North R. A. (2006). P2X receptors as cell-surface ATP sensors in health and disease. Nature 442 (7102), 527–532. 10.1038/nature04886 16885977

[B53] Khalid M. Brisson L. Tariq M. Hao Y. Guibon R. Fromont G. (2017). Carcinoma-specific expression of P2Y11 receptor and its contribution in ATP-induced purinergic signalling and cell migration in human hepatocellular carcinoma cells. Oncotarget 8 (23), 37278–37290. 10.18632/oncotarget.16191 28418839 PMC5514908

[B54] Kim M. J. Kim H. S. Lee S. H. Yang Y. Lee M. S. Lim J. S. (2014). NDRG2 controls COX-2/PGE₂-mediated breast cancer cell migration and invasion. Mol. Cell 37 (10), 759–765. 10.14348/molcells.2014.0232 PMC4213768 25256221

[B55] Kloss L. Dollt C. Schledzewski K. Krewer A. Melchers S. Manta C. (2019). ADP secreted by dying melanoma cells mediates chemotaxis and chemokine secretion of macrophages via the purinergic receptor P2Y12. Cell Death Dis. 10 (10), 760 10.1038/s41419-019-2010-6 31591378 PMC6779894

[B56] Kuang W. Deng Q. Deng C. Li W. Shu S. Zhou M. (2017). Hepatocyte growth factor induces breast cancer cell invasion via the PI3K/Akt and p38 MAPK signaling pathways to up-regulate the expression of COX2. Am. J. Transl. Res. 9 (8), 3816–3826. 28861172 PMC5575195

[B57] Lala P. K. Nandi P. Majumder M. (2018). Roles of prostaglandins in tumor-associated lymphangiogenesis with special reference to breast cancer. Cancer Metastasis Rev. 37 (2-3), 369–384. 10.1007/s10555-018-9734-0 29858743

[B58] Lara R. Adinolfi E. Harwood C. A. Philpott M. Barden J. A. Di Virgilio F. (2020). P2X7 in cancer: from molecular mechanisms to therapeutics. Front. Pharmacol. 11, 793 10.3389/fphar.2020.00793 32581786 PMC7287489

[B59] Lasa M. Mahtani K. R. Finch A. Brewer G. Saklatvala J. Clark A. R. (2000). Regulation of cyclooxygenase 2 mRNA stability by the mitogen-activated protein kinase p38 signaling cascade. Mol. Cell Biol. 20 (12), 4265–4274. 10.1128/mcb.20.12.4265-4274.2000 10825190 PMC85794

[B60] Lecciso M. Ocadlikova D. Sangaletti S. Trabanelli S. De Marchi E. Orioli E. (2017). ATP release from chemotherapy-treated dying leukemia cells elicits an immune suppressive effect by increasing regulatory T cells and tolerogenic dendritic cells. Front. Immunol. 8, 1918 10.3389/fimmu.2017.01918 29312358 PMC5744438

[B61] Li G. Yang T. Yan J. (2002). Cyclooxygenase-2 increased the angiogenic and metastatic potential of tumor cells. Biochem. Biophys. Res. Commun. 299 (5), 886–890. 10.1016/s0006-291x(02)02707-9 12470662

[B62] Li W. H. Qiu Y. Zhang H. Q. Liu Y. You J. F. Tian X. X. (2013). P2Y2 receptor promotes cell invasion and metastasis in prostate cancer cells. Br. J. Cancer 109 (6), 1666–1675. 10.1038/bjc.2013.484 23969730 PMC3776994

[B63] Liang C. C. Park A. Y. Guan J. L. (2007). *In vitro* scratch assay: a convenient and inexpensive method for analysis of cell migration *in vitro* . Nat. Protoc. 2 (2), 329–333. 10.1038/nprot.2007.30 17406593

[B64] Lili W. Yun L. Tingran W. Xia W. Yanlei S. (2019). P2RX7 functions as a putative biomarker of gastric cancer and contributes to worse prognosis. Exp. Biol. Med. 244 (9), 734–742. 10.1177/1535370219846492 PMC6567584 31042071

[B65] Liu C. L. Chen M. J. Lin J. C. Lin C. H. Huang W. C. Cheng S. P. (2019). Doxorubicin promotes migration and invasion of breast cancer cells through the upregulation of the RhoA/MLC pathway. J. Breast Cancer 22 (2), 185–195. 10.4048/jbc.2019.22.e22 31281722 PMC6597404

[B66] Ma X. Pan X. Wei Y. Tan B. Yang L. Ren H. (2016). Chemotherapy-induced uridine diphosphate release promotes breast cancer metastasis through P2Y6 activation. Oncotarget 7 (20), 29036–29050. 10.18632/oncotarget.8664 27074554 PMC5045376

[B67] Mandapathil M. Boduc M. Roessler M. Güldner C. Walliczek-Dworschak U. Mandic R. (2018). Ectonucleotidase CD39 expression in regional metastases in head and neck cancer. Acta Otolaryngol. 138 (4), 428–432. 10.1080/00016489.2017.1405278 29172836

[B68] Martins I. Tesniere A. Kepp O. Michaud M. Schlemmer F. Senovilla L. (2009). Chemotherapy induces ATP release from tumor cells. Cell Cycle 8 (22), 3723–3728. 10.4161/cc.8.22.10026 19855167

[B69] Minghetti L. (2007). Role of COX-2 in inflammatory and degenerative brain diseases. Subcell. Biochem. 42, 127–141. 10.1007/1-4020-5688-5_5 17612048

[B70] Moesta A. K. Li X. Y. Smyth M. J. (2020). Targeting CD39 in cancer. Nat. Rev. Immunol. 20 (12), 739–755. 10.1038/s41577-020-0376-4 32728220

[B71] Nakanishi M. Rosenberg D. W. (2013). Multifaceted roles of PGE2 in inflammation and cancer. Semin. Immunopathol. 35 (2), 123–137. 10.1007/s00281-012-0342-8 22996682 PMC3568185

[B72] Nakanishi Y. Kamijo R. Takizawa K. Hatori M. Nagumo M. (2001). Inhibitors of cyclooxygenase-2 (COX-2) suppressed the proliferation and differentiation of human leukaemia cell lines. Eur. J. Cancer 37 (12), 1570–1578. 10.1016/s0959-8049(01)00160-5 11506967

[B73] Parvathenani L. K. Tertyshnikova S. Greco C. R. Roberts S. B. Robertson B. Posmantur R. (2003). P2X7 mediates superoxide production in primary microglia and is up-regulated in a transgenic mouse model of Alzheimer’s disease. J. Biol. Chem. 278 (15), 13309–13317. 10.1074/jbc.M209478200 12551918

[B74] Pavlović N. Kopsida M. Gerwins P. Heindryckx F. (2020). Inhibiting P2Y12 in macrophages induces endoplasmic reticulum stress and promotes an anti-tumoral phenotype. Int. J. Mol. Sci. 21 (21), 8177 10.3390/ijms21218177 PMC7672568 33142937

[B75] Pegoraro A. Orioli E. De Marchi E. Salvestrini V. Milani A. Di Virgilio F. (2020). Differential sensitivity of acute myeloid leukemia cells to daunorubicin depends on P2X7A versus P2X7B receptor expression. Cell Death Dis. 11 (10), 876 10.1038/s41419-020-03058-9 33071281 PMC7569086

[B76] Qin J. Zhang Z. Fu Z. Ren H. Liu M. Qian M. (2020). The UDP/P2y6 axis promotes lung metastasis of melanoma by remodeling the premetastatic niche. Cell. Mol. Immunol. 17 (12), 1269–1271. 10.1038/s41423-020-0392-0 32144377 PMC7784845

[B77] Qiu Y. Li W. H. Zhang H. Q. Liu Y. Tian X. X. Fang W. G. (2014). P2X7 mediates ATP-driven invasiveness in prostate cancer cells. PLoS One 9 (12), e114371 10.1371/journal.pone.0114371 25486274 PMC4259308

[B78] Ristimäki A. Sivula A. Lundin J. Lundin M. Salminen T. Haglund C. (2002). Prognostic significance of elevated cyclooxygenase-2 expression in breast cancer. Cancer Res. 62 (3), 632–635. 11830510

[B79] Roomi M. W. Kalinovsky T. Rath M. Niedzwiecki A. (2017). Modulation of MMP-2 and MMP-9 secretion by cytokines, inducers and inhibitors in human glioblastoma T-98G cells. Oncol. Rep. 37 (3), 1907–1913. 10.3892/or.2017.5391 28112361

[B80] Schneider G. Glaser T. Lameu C. Abdelbaset-Ismail A. Sellers Z. P. Moniuszko M. (2015). Extracellular nucleotides as novel, underappreciated pro-metastatic factors that stimulate purinergic signaling in human lung cancer cells. Mol. Cancer 14, 201 10.1186/s12943-015-0469-z 26597723 PMC4657356

[B81] Secchiero P. Barbarotto E. Gonelli A. Tiribelli M. Zerbinati C. Celeghini C. (2005). Potential pathogenetic implications of cyclooxygenase-2 overexpression in B chronic lymphoid leukemia cells. Am. J. Pathol. 167 (6), 1599–1607. 10.1016/S0002-9440(10)61244-8 16314473 PMC1613188

[B82] Shi K. Queiroz K. C. Stap J. Richel D. J. Spek C. A. (2013). Protease-activated receptor-2 induces migration of pancreatic cancer cells in an extracellular ATP-dependent manner. J. Thromb. Haemostasis 11 (10), 1892–1902. 10.1111/jth.12361 23899344

[B83] Singh B. Berry J. A. Shoher A. Ayers G. D. Wei C. Lucci A. (2007). COX-2 involvement in breast cancer metastasis to bone. Oncogene 26 (26), 3789–3796. 10.1038/sj.onc.1210154 17213821

[B84] Soleimani A. Taghizadeh E. Shahsavari S. Amini Y. Rashidpour H. Azadian E. (2019). CD73; a key ectonucleotidase in the development of breast cancer: recent advances and perspectives. J. Cell. Physiol. 234 (9), 14622–14632. 10.1002/jcp.28187 30693504

[B85] Toth M. Sohail A. Fridman R. (2012). Assessment of gelatinases (MMP-2 and MMP-9) by gelatin zymography. Methods Mol. Biol. 878, 121–135. 10.1007/978-1-61779-854-2_8 22674130

[B86] Tumilowicz J. J. Nichols W. W. Cholon J. J. Greene A. E. (1970). Definition of a continuous human cell line derived from neuroblastoma. Cancer Res. 30 (8), 2110–2118. 5459762

[B87] Ulrich C. M. Bigler J. Potter J. D. (2006). Non-steroidal anti-inflammatory drugs for cancer prevention: promise, perils and pharmacogenetics. Nat. Rev. Cancer 6 (2), 130–140. 10.1038/nrc1801 16491072

[B88] Vultaggio-Poma V. Sarti A. C. Di Virgilio F. (2020). Extracellular ATP: a feasible target for cancer therapy. Cells 9 (11). 10.3390/cells9112496 PMC7698494 33212982

[B89] Wan L. Pantel K. Kang Y. (2013). Tumor metastasis: moving new biological insights into the clinic. Nat. Med. 19 (11), 1450–1464. 10.1038/nm.3391 24202397

[B90] Wang D. DuBois R. N. (2015). Immunosuppression associated with chronic inflammation in the tumor microenvironment. Carcinogenesis 36 (10), 1085–1093. 10.1093/carcin/bgv123 26354776 PMC5006153

[B91] Wang M. T. Honn K. V. Nie D. (2007). Cyclooxygenases, prostanoids, and tumor progression. Cancer Metastasis Rev. 26 (3-4), 525–534. 10.1007/s10555-007-9096-5 17763971

[B92] Wiedemar N. Hauser D. A. Mäser P. (2020). 100 Years of suramin. Antimicrob. Agents Chemother. 64 (3). 10.1128/AAC.01168-19 PMC7038244 31844000

[B93] Wun T. McKnight H. Tuscano J. M. (2004). Increased cyclooxygenase-2 (COX-2): a potential role in the pathogenesis of lymphoma. Leuk. Res. 28 (2), 179–190. 10.1016/s0145-2126(03)00183-8 14654083

[B94] Yan M. Rerko R. M. Platzer P. Dawson D. Willis J. Tong M. (2004). 15-Hydroxyprostaglandin dehydrogenase, a COX-2 oncogene antagonist, is a TGF-beta-induced suppressor of human gastrointestinal cancers. Proc. Natl. Acad. Sci. U.S.A. 101 (50), 17468–17473. 10.1073/pnas.0406142101 15574495 PMC536023

[B95] Yang L. Cranson D. Trinkaus-Randall V. (2004). Cellular injury induces activation of MAPK via P2Y receptors. J. Cell. Biochem. 91 (5), 938–950. 10.1002/jcb.10774 15034929

[B96] Zhang Y. Cheng H. Li W. Wu H. Yang Y. (2019). Highly-expressed P2X7 receptor promotes growth and metastasis of human HOS/MNNG osteosarcoma cells via PI3K/Akt/GSK3beta/beta-catenin and mTOR/HIF1alpha/VEGF signaling. Int. J. Cancer 145 (4), 1068–1082. 10.1002/ijc.32207 30761524 PMC6618011

